# Kinesin-binding protein remodels the kinesin motor to prevent microtubule binding

**DOI:** 10.1126/sciadv.abj9812

**Published:** 2021-11-19

**Authors:** April L. Solon, Zhenyu Tan, Katherine L. Schutt, Lauren Jepsen, Sarah E. Haynes, Alexey I. Nesvizhskii, David Sept, Jason Stumpff, Ryoma Ohi, Michael A. Cianfrocco

**Affiliations:** 1 Department of Cell and Developmental Biology, University of Michigan, Ann Arbor, MI, USA.; 2 Department of Biophysics, University of Michigan, Ann Arbor, MI, USA.; 3 Life Sciences Institute, University of Michigan, Ann Arbor, MI, USA.; 4 Department of Molecular Physiology and Biophysics, University of Vermont, Burlington, VT, USA.; 5 Department of Biomedical Engineering, University of Michigan, Ann Arbor, MI, USA.; 6 Department of Pathology, University of Michigan, Ann Arbor, MI, USA.; 7 Department of Computational Medicine and Bioinformatics, University of Michigan, Ann Arbor, MI, USA.; 8 Department of Biological Chemistry, University of Michigan, Ann Arbor, MI, USA.

## Abstract

Kinesins are regulated in space and time to ensure activation only in the presence of cargo. Kinesin-binding protein (KIFBP), which is mutated in Goldberg-Shprintzen syndrome, binds to and inhibits the catalytic motor heads of 8 of 45 kinesin superfamily members, but the mechanism remains poorly defined. Here, we used cryo–electron microscopy and cross-linking mass spectrometry to determine high-resolution structures of KIFBP alone and in complex with two mitotic kinesins, revealing structural remodeling of kinesin by KIFBP. We find that KIFBP remodels kinesin motors and blocks microtubule binding (i) via allosteric changes to kinesin and (ii) by sterically blocking access to the microtubule. We identified two regions of KIFBP necessary for kinesin binding and cellular regulation during mitosis. Together, this work further elucidates the molecular mechanism of KIFBP-mediated kinesin inhibition and supports a model in which structural rearrangement of kinesin motor domains by KIFBP abrogates motor protein activity.

## Teaser

Kinesin-binding protein inhibits kinesin activity through structural alterations of the kinesin motor domain.

## INTRODUCTION

Kinesins comprise a superfamily of microtubule-based motor proteins that play essential roles in virtually every aspect of cell physiology, including mitotic spindle assembly, regulation of microtubule dynamics, ciliogenesis, and transportation of cargoes throughout the cell (*1*–*4*). A signature protein fold shared among all members of the kinesin superfamily is a catalytic “motor” domain. The kinesin motor domain contains binding sites for both microtubules and adenosine 5′-triphosphate (ATP), enabling these proteins to convert energy from ATP hydrolysis into mechanical force (*5*). In most kinesin motors, this catalytic cycle powers motility of the proteins along microtubule tracks. While the motor domain exhibits structural and high sequence conservation among the superfamily, sequence differences imbue each kinesin with unique characteristics and are responsible for diversifying motor functions within the cell. In addition, the nonmotor regions of different kinesin family members have diverged to confer specificity for cargo binding and regulation.

Kinesins are regulated at many levels to ensure that they become activated at the right time and place. Autoinhibition, wherein kinesins adopt a conformation that prevents microtubule binding (*6*–*8*), as well as sequestration within the nucleus (*9*–*14*) and cell cycle–dependent protein expression (*15*) are common strategies to prevent untimely motor-track interactions. Kinesins are also regulated by posttranslational modifications, e.g., phosphorylation, which can serve to activate microtubule binding (*16*–*18*). Last, kinesin-interacting proteins such as adaptor proteins and light chains, and their phosphorylation, can regulate the ability of transport kinesins to engage cargo (*1*, *19*–*24*) or target them to specific locations within the cell (*25*, *26*). KIFBP, a unique class of kinesin-binding protein, has emerged as an important negative regulator of a subset of kinesin motors (*27*, *28*).

KIFBP was found as a disease-causing gene associated with the neurological disorder Goldberg-Shprintzen syndrome [GOSHS (*29*–*31*)], an autosomal disease characterized by facial dysmorphism, mental retardation, and congenital heart disease [OMIM (Online Mendelian Inheritance in Man) #609460]. In mice and zebrafish, loss of KIFBP function leads to neuronal migration and maturation defects in the developing brain (*32*, *33*). Emerging data demonstrate a compelling role for KIFBP in regulating motor-microtubule interactions for 8 of the 45 kinesin motors encoded by the human genome. KIFBP interacts directly with the motor head of Kinesin-2 (KIF3A), Kinesin-3 (KIF1A, KIF1B, KIF1C, KIF13B, and KIF14), Kinesin-8 (KIF18A), and Kinesin-12 (KIF15) family members, resulting in inhibition of motor-microtubule binding both in vitro and in cells (*27*, *28*). How the regulation of kinesin motors by KIFBP is linked to specific biological processes is largely unexplored, although neuronal microtubule dynamics appear to be controlled through KIFBP-dependent regulation of KIF18A (*27*). Moreover, recent work has shown that KIFBP is critical for ensuring proper mitotic spindle assembly by regulating the mitotic kinesins KIF15 and KIF18A (*28*).

Recently, a 4.6-Å structure for KIFBP and a 6.9-Å structure for KIFBP bound to KIF15 were reported (*34*). These studies indicated that KIFBP alters the KIF15 kinesin motor to prevent microtubule binding (*34*). Despite the advances afforded by this study, many open questions remain. First, the resolution of the published structures did not fully define the KIFBP-interaction interface with KIF15 given the large degree of uncertainty for the atomic model. For example, at the resolutions reported, the atomic models may have incorrect helical placement or helical registries. Without an accurate atomic model of KIFBP, the molecular mechanism of kinesin regulation by KIFBP remains unclear. Second, the generality of the previously proposed inhibition mechanism is unknown. The earlier work also included an analysis of KIFBP in complex with KIF1A, but the low resolution of the KIFBP-KIF1A complex and heterogeneity of binding poses did not allow firm conclusions regarding the binding mechanism. Third, the previous study did not address the importance of residue-residue contacts between KIFBP and kinesin motors (KIF15 and KIF1A) in the context of the specific biological processes in which these motors participate.

To understand how KIFBP engages kinesin motors, we leveraged an interdisciplinary approach to generate a high-confidence atomic model of the KIFBP–kinesin motor interface by combining cryo–electron microscopy (cryo-EM) with cross-linking mass spectrometry (XL-MS). We show that KIFBP is a tandem repeat protein constructed of nine helix pairs that assemble into a solenoid-like structure. When complexed with KIF15 and KIF18A, three helices in KIFBP [helical pair 4a/b (HP4a/b)–HP5] associate closely with the kinesin α4 helix, an interaction that requires a 15-Å displacement of α4 from its resting position. Using molecular dynamics (MD) simulations, we find that kinesin α4 is immobile when a motor head is not bound to KIFBP, suggesting that allosteric changes drive the repositioning of α4 required for binding HP4a/b-HP5. Using our high-confidence KIFBP:kinesin atomic model, we identify two regions in KIFBP that are responsible for the interaction in vitro and show that mutations in these regions disable the ability of KIFBP to regulate KIF15 and KIF18A during mitosis. Collectively, our work describes the molecular mechanism of KIFBP-mediated inhibition of KIF15 and KIF18A via binding to and stabilizing a conformation of the kinesin motor head that is incompatible with microtubule binding.

## RESULTS

### KIFBP adopts a solenoid structure composed of TPR motifs

**Fig. 1.**
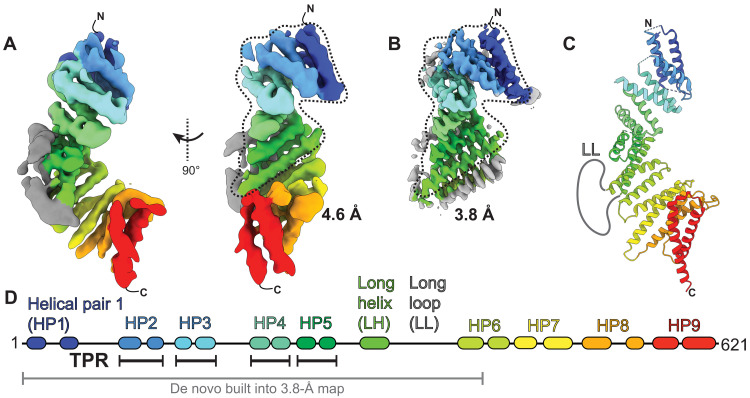
KIFBP adopts a solenoid structure composed of TPR motifs. (**A**) Overview of KIFBP structure at 4.6 Å. Dotted lines indicate the masked region for the higher-resolution KIFBP core. (**B**) Structure of KIFBP core at 3.8 Å. (**C**) Combined atomic model of KIFBP. (**D**) Structural features and nomenclature for KIFBP.

To determine an atomic model of KIFBP, we used cryo-EM to determine the overall structure of KIFBP and a higher-resolution structure of the N terminus of KIFBP (Fig. 1 and movie S1). Reconstructions of the full KIFBP molecule at 4.6 Å showed that KIFBP is almost entirely α-helical, having nine HPs along with one long helix throughout the 621–amino acid sequence, similar to previous reports (*34*) (Fig. 1A, figs. S1 and S2, and table S1). The α helices are arranged into a right-handed superhelical twist, giving KIFBP an appearance analogous to other tetratricopeptide repeat (TPR) proteins (*35*).

Given that the resolution of 4.6 Å is not sufficient to build an atomic model, we wanted to improve the resolution of our reconstruction. To this end, we performed masking and three-dimensional (3D) classification on the N-terminal two-thirds of KIFBP to obtain a higher-resolution structure at 3.8 Å (Fig. 1B). We could unambiguously identify amino acid side chains at this resolution, allowing us to construct an atomic model for amino acids 5 to 403 (Fig. 1C, figs. S1 and S3 to S5, and table S2). Our atomic model provides a high-confidence positioning of KIFBP residues, allowing us to map the structure onto the KIFBP sequence (Fig. 1D), confirming α-helical positions, and showing the locations of loops connecting HPs throughout the structure.

### KIFBP inhibits KIF15 microtubule binding and remodels the motor domain of KIF15

After determining the structure of KIFBP alone, we used cryo-EM to determine the structure of KIFBP bound to KIF15. To prepare cryo-EM samples, we incubated the purified KIF15 motor domain (amino acids 1 to 375) with KIFBP and subjected the sample to size exclusion chromatography in the presence of ATP (fig. S6). SDS–polyacrylamide gel electrophoresis (SDS-PAGE) analysis determined that KIF15 comigrated with KIFBP in a 1:1 complex, and fractions containing the complex were used for cryo-EM sample preparation.

**Fig. 2.**
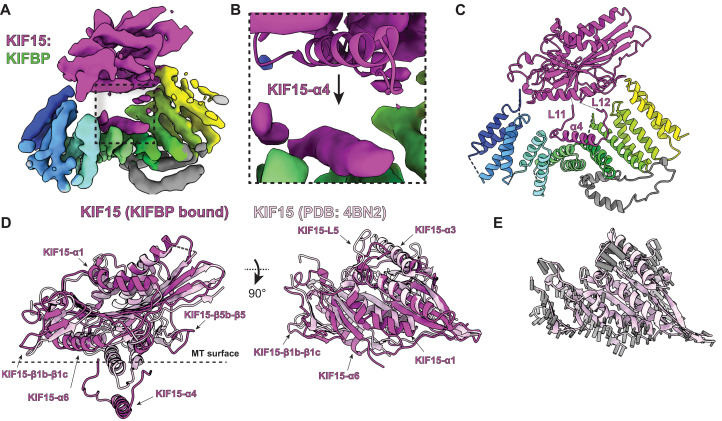
KIFBP stabilizes KIF15 in a conformation that blocks microtubule binding. (**A**) KIF15:KIFBP reconstruction. (**B**) Zoom-in on additional density present in KIF15:KIFBP reconstruction (purple) alongside docked crystal structure [Protein Data Bank (PDB): 4BN2] (*37*). (**C**) Atomic model of KIF15:KIFBP. (**D**) Superposition of KIF15 bound to KIFBP (dark purple) with apo-KIF15 (light purple) (PDB: 4BN2) (*37*) relative to KIF15-α2. Structural elements that differ are indicated by arrows. MT, microtubule. (**E**) Vectors (gray) calculated from Cα differences between KIF15 (KIFBP-bound) and KIF15 superimposed on the apo KIF15 crystal structure (PDB: 4BN2) (*37*).

We used cryo-EM to determine a ~4.8-Å-resolution structure of KIFBP bound to KIF15 (Fig. 2A, figs. S7 and S8, table S3, and movie S1). At this resolution, we could unambiguously identify the regions of density corresponding to KIFBP in addition to the motor domain of KIF15 (Fig. 2A). Unexpectedly, when we docked the structure of KIF15 into the reconstruction, we noticed that the α4 helix of KIF15 was missing. Instead, we noticed the presence of an additional α-helical density within a cleft of KIFBP, suggesting the displacement of KIF15-α4 into this cleft during complex formation (Fig. 2B). In the structure, KIFBP occupies the microtubule-binding surface of KIF15, sterically blocking access to the microtubules by KIF15.

To understand how KIFBP affected the overall architecture of KIF15, we used both manual building and Rosetta comparative modeling (*36*) to develop a model for the KIFBP-engaged KIF15 motor (Fig. 2C). Our analysis of KIFBP:KIF15 revealed structural rearrangements of the KIF15 motor by KIFBP to disrupt KIF15’s microtubule-binding interface. The most notable structural change involved the repositioning of KIF15-α4 away from the kinesin motor domain, placing KIF15-α4 15 Å away from the location found in the crystal structure of KIF15 (*37*). Notably, kinesin motors require the α4 helix for engaging microtubules during the kinesin mechanochemical cycle (*38*). The adjoining loops on each side of KIF15-α4, Loop-11 (“KIF15-L11”) and Loop-12 (“KIF15-L12”), accommodated the repositioning of KIF15-α4 by facilitating the extension of KIF15-α4 away from the body of the motor domain (Fig. 2C). Whereas KIF15-L12 remains extended in solution, KIF15-L11 is positioned away from the motor domain and binds along KIFBP-HP4a.

In addition to seeing changes in KIF15-L11, KIF15-α4, and KIF15-L12, we saw that the overall structure of KIF15 adopted a more open conformation (Fig. 2, D and E). The structure showed the shift of α helices KIF15-α1, KIF15-α3, and KIF15-α6 away from the core of the motor. We observed large movements for β-strand pairs KIF15-β1b-β1c and KIF15-β5b-β5 in addition to loop KIF15-L5. These changes indicate that KIFBP stabilizes several structural changes in KIF15 to block microtubule binding. Thus, KIFBP blocks microtubule binding by sterically preventing microtubule interaction in addition to allosterically altering the KIF15 motor.

### KIFBP binds to KIF15-α4 in a distinct manner relative to αβ-tubulin

**Fig. 3.**
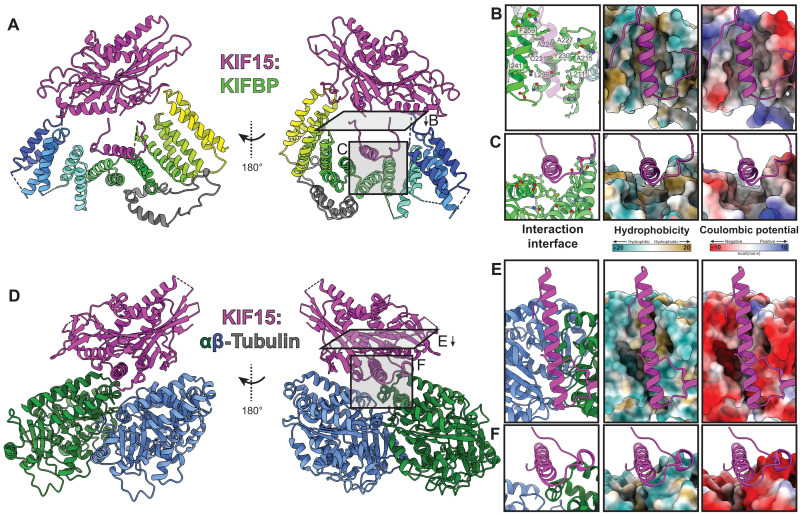
KIFBP binds to KIF15-α4 in a distinct manner relative to tubulin. Comparison of KIFBP- and αβ-tubulin–bound KIF15. (**A**) KIF15:KIFBP atomic model. Right: Gray rectangles indicate viewing directions for (B) and (C). (**B** and **C**) Top and side views of KIF15-α4 interface (left), hydrophobicity (center), and Coulombic potential (right). KIFBP-binding residues for KIF15-α4 are shown in ball-and-stick mode, and hydrophobic residues are labeled in (B). (**D**) KIF15:αβ-tubulin structure (PDB: 6ZPI) (*34*) shown relative to KIF15:KIFBP (A). Right: Gray rectangles indicate viewing directions for (E) and (F). (**E** and **F**) Top and side views of KIF15-α4 interface (left), hydrophobicity (center), and Coulombic potential (right).

Given that KIFBP binds along the microtubule-binding interface of KIF15, we sought to compare the interaction interface between KIF15:KIFBP and KIF15:αβ-tubulin (*34*). First, we noticed that the length of the α4 helix is shorter for KIF15-KIFBP compared to the microtubule-engaged α4 helix (Fig. 3, A to F). The length of α4 in KIFBP:KIF15 is similar to the crystal structure of KIF15 when not bound to microtubules (*37*). Second, KIF15-L11 is bent relative to α4 at an angle of ~120° (Fig. 3B), whereas KIF15-L11 on the microtubule adopts a helical structure to extend the length of α4 (Fig. 3E) (*34*). These two observations indicate that KIFBP holds KIF15-α4 in a conformation that is incompatible with microtubule binding.

Comparing the hydrophobicity and electrostatic charge surfaces on KIFBP versus αβ-tubulin shows that KIFBP binds KIF15-α4 via hydrophilic and hydrophobic helices (Fig. 3, B, C, E, and F, and movie S1). The strong electrostatic nature of αβ-tubulin results in minimal hydrophobic residues contributing to KIF15 binding. Unlike β-tubulin, KIFBP uses a composite binding site stretching across three helices to bind both hydrophobic and polar residues to interact with KIF15-α4. Comparing the overall hydrophobicity and charge distribution indicates that KIFBP binds KIF15-α4 in a manner distinct from αβ-tubulin.

### KIFBP engages the microtubule-binding interface of KIF15 using multiple contact points

**Fig. 4.**
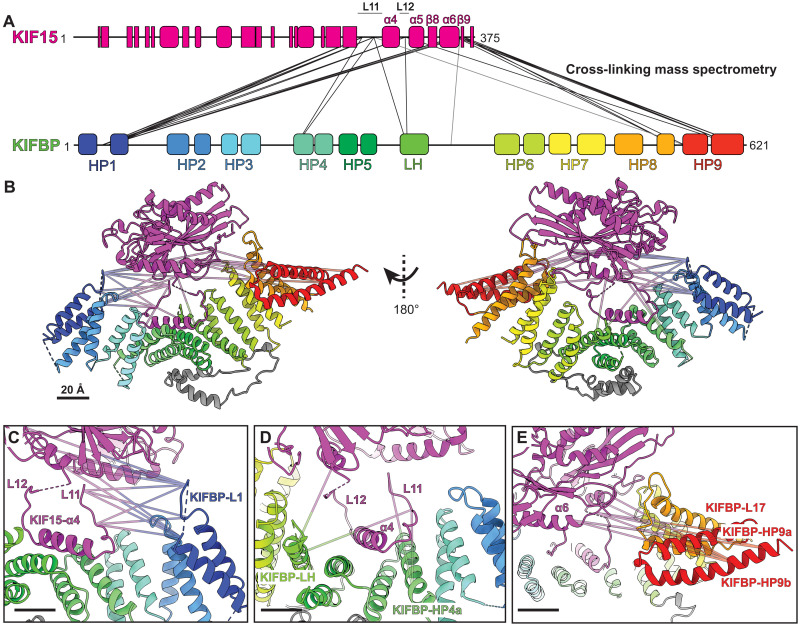
KIFBP physically contacts multiple sites along the microtubule-binding interface of KIF15. (**A**) Schematic representing the location of identified cross-links between the KIF15 motor domain (top) and KIFBP (bottom). Secondary structure elements of the two proteins are represented by rectangles (β sheets), rounded rectangles (α helices), and lines (unstructured regions). (**B**) Cross-links shown in (A) have been superimposed on the cryo-EM structure of KIF15:KIFBP. (**C**) Zoomed-in view of the cross-links between KIFBP-L1 and KIF15. (**D**) Zoomed-in view of the cross-links between KIFBP-HP4a and KIFBP-LH and KIF15. (**E**) Zoomed-in view of the cross-links between KIFBP-L17 and KIFBP-HP9a/b and KIF15. Scale bars, 20 Å.

To obtain further insight into the regions of KIFBP and KIF15 that interact with each other, we performed XL-MS. Recombinant KIFBP and KIF15 (1 to 375) were incubated with the 11-Å lysine-targeting cross-linker BS3, digested with trypsin, and analyzed using tandem mass spectrometry (MS/MS). We identified cross-linked peptides using pLink software (see Materials and Methods). We present all high-confidence cross-links between KIFBP and KIF15 peptides (*e* value of >0.05) in table S5 and have displayed them on the primary and secondary structures of KIFBP:KIF15 as well (Fig. 4, A and B, and movie S1).

We observed the highest density of cross-links between three residues of KIFBP-L1 (K26, K30, and K36) and the microtubule-binding interface of KIF15 (K273, K283, K319, and K361) (Fig. 4, A and C). These same KIF15 residues also cross-linked to regions in the middle of KIFBP [HP4a, LH (long helix), and LL (long loop)] (Fig. 4D) and toward the C terminus (L17, HP9a, and HP9b) (Fig. 4E). Two KIF15 residues that cross-linked multiple KIFBP sites (K273 and K283) are located in KIF15-L11, adjacent to KIF15-α4. In addition, residues KIF15-K273 and KIF15-K319 form the kinesin motor microtubule-binding interface (*34*). The high density of cross-links involving these KIF15 residues supports a mechanism of inhibition where KIFBP directly binds the microtubule-binding domain of kinesins, occluding interactions with the microtubule lattice.

KIFBP:KIF15 cross-links span nearly the entire length of KIFBP to bind the KIF15 microtubule-binding surface. When superimposed onto the KIFBP:KIF15 structure (Fig. 4, B to E), these cross-links bridge distances greater than the 11-Å BS3 can reach. This suggests that the cross-linked regions of KIFBP may associate transiently with the microtubule-binding interface of KIF15 at different time points during complex formation (see Discussion).

### KIFBP inhibits KIF18A via a similar mechanism as KIF15

After characterizing how KIFBP inhibits KIF15 (kinesin-12 family), we next aimed to establish whether KIFBP uses the same mode of inhibition for a kinesin motor from a different kinesin family, KIF18A (kinesin-8 family). To determine how KIFBP inhibits KIF18A, we first purified recombinant KIF18A (1–363) motor domain, incubated KIF18A with KIFBP, and performed size exclusion chromatography to confirm the formation of a 1:1 complex (fig. S9). After preparing cryo-EM grids with the complex, we obtained 2D class averages that appeared similar in shape and features as seen previously for KIF15 (fig. S10), further confirming the formation of a KIFBP:KIF18A complex.

**Fig. 5.**
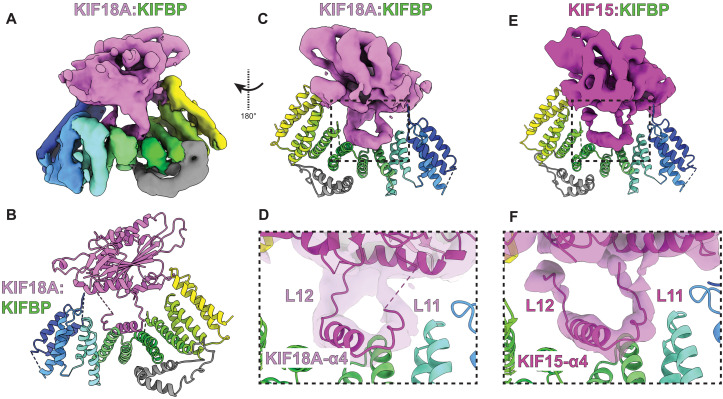
KIFBP inhibits KIF18A via a similar mechanism as KIF15. (**A**) Cryo-EM reconstruction of KIF18A:KIFBP. (**B**) Atomic model of KIF18A:KIFBP. (**C**) Segmented KIF18A density shown alongside KIFBP atomic model, rotated 180° from (A). (**D**) Zoomed-in view of KIF18A density and model on KIFBP interface. (**E**) Segmented KIF15 density shown in a similar orientation as (C). (**F**) Zoomed-in view of KIF15:KIFBP interface.

After performing further single-particle analysis, the cryo-EM structure of KIFBP:KIF18A revealed that KIFBP binds KIF18A similar to KIF15 (Fig. 5, A and B, fig. S11, table S4, and movie S1). In the structure, KIFBP N- and C-terminal domains engage both sides of the motor domain, while KIF18A-α4 is displaced away from the motor into the central cavity of KIFBP. To highlight the similarities between KIFBP engagement of KIF15 and KIF18A, we segmented the motor density from either KIFBP:KIF18A (Fig. 5, C and D) or KIFBP:KIF15 (Fig. 5, E and F). This comparison shows for both motors that (i) α4 is held within the central cleft of KIFBP, (ii) Loop-11 and Loop-12 are extended away from the motor, and (iii) KIFBP-L11 adopts a curved shape as it makes a ~120° turn to follow helix KIFBP-HP4a within the KIFBP cleft. Thus, KIFBP stabilization of kinesin α4 helix away from the motor is a shared mode of kinesin inhibition by KIFBP for KIF18A and KIF15.

### KIFBP uses Loop-1 and Loop-14 to bind kinesin in vitro

**Fig. 6.**
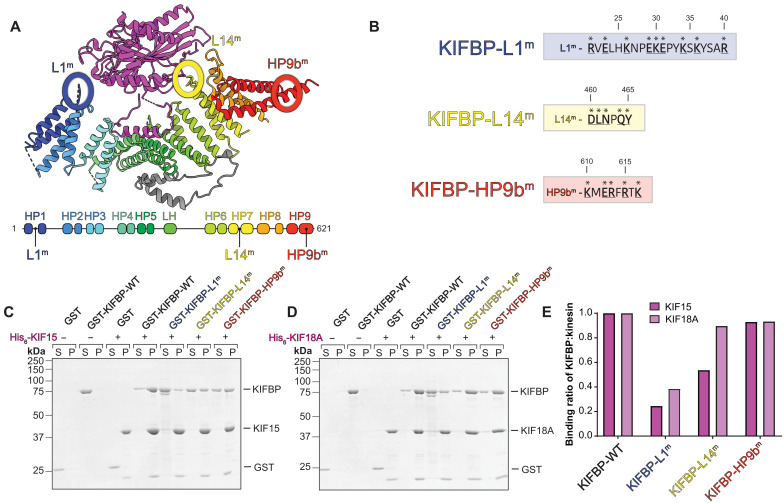
KIFBP uses Loop-1 and Loop-14 to bind kinesins in vitro. (**A**) Schematic showing the locations of the three mutations on the cryo-EM structure of KIF15:KIFBP (top) and the secondary structure of KIFBP (bottom). Mutations were made to Loop-1, Loop-14, and HP9b of KIFBP. (**B**) Mutated residues in each mutant are indicated with asterisks; residue position is indicated above each sequence. (**C** and **D**) Representative Coomassie gels showing results of in vitro pull-down assays where KIFBP proteins were pulled down by either KIF15 (C) or KIF18A (D). Supernatant and pellet samples, as well as the presence or absence of kinesin, are indicated at the top of each gel. (**E**) Quantifications of pull-down assays shown in (C) and (D) are graphed as the ratio of each KIFBP protein to KIF15 or KIF18A in the pellet.

Our structural data and XL-MS results identified multiple KIFBP-motor interactions that may be important for robust binding and inhibition of motor activity. In particular, our XL-MS results revealed that the most extensively cross-linked residues in KIFBP occurred within Loop-1 and the C-terminal helix pairs of KIFBP (Fig. 4A and table S5). Our cryo-EM structure of KIFBP complexed with the motor domains of both KIF15 and KIF18A supports a prominent role for Loop-1 in motor engagement. We therefore targeted both KIFBP-L1 and KIFBP-HP9b for mutagenesis, selecting positively and negatively charged residues in those regions and substituting them with alanine or glycine residues (Fig. 6B). We also constructed a third KIFBP mutant by mutating residues 460 to 465 in KIFBP-L14 to alanine (Fig. 6B) because of the proximity of KIFBP-L14 to KIF15 and previous work indicating that it is important for kinesin motor binding (*34*). All proteins were purified for in vitro binding studies (fig. S12).

First, we tested the ability of these three KIFBP mutants to bind the motor domains of KIF15 and KIF18A in vitro. We performed in vitro pull-down assays using hexahistidine-tagged motor domains of either KIF15 (1 to 375) or KIF18A (1 to 363) immobilized on nickel resin and analyzed the ratio of recombinant wild-type (WT) or mutant KIFBP to kinesin motor domain in the pellet. We used glutathione *S*-transferase (GST) as a negative control, which showed little to no nonspecific interaction with nickel resin or immobilized kinesin. When incubated with KIF15, we observed a fourfold reduction in binding of the KIFBP-L1^m^ mutant and a twofold reduction in binding of the KIFBP-L14^m^ mutant compared to KIFBP-WT, indicating that mutations in these regions abrogate the ability of KIFBP to interact with the motor robustly. Unexpectedly, KIFBP-HP9b^m^ showed no difference in binding compared to KIFBP-WT, suggesting that the charged residues that mutated are not essential for motor binding. Thus, although both Loop-1 and the C terminus of KIFBP were implicated as potentially important for binding in our XL-MS experiment (Fig. 4, C and E), mutations to only one of these regions, Loop-1, biochemically affected binding.

Next, we repeated pull-down assays with KIF18A immobilized on the resin and analyzed the binding of the same panel of mutants. Similar to KIF15, the KIFBP-L1^m^ mutant showed a threefold reduction in binding compared to KIFBP-WT. Intriguingly, the KIFBP-L14^m^ mutant showed only a slight 10% reduction in binding to KIF18A compared to KIFBP-WT, in contrast to the 50% decrease in binding to KIF15.

### Mutations in KIFBP-L1 and KIFBP-L14 disrupt the regulation of mitotic kinesins

**Fig. 7.**
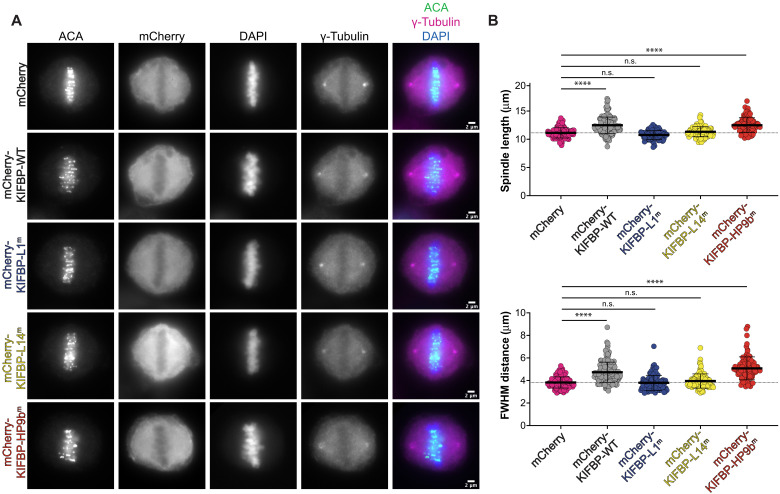
Mutations in KIFBP-L1 and KIFBP-L14 diminish KIFBP-mediated regulation of spindle length and chromosome alignment during mitosis. (**A**) MG132 (*N*-carbobenzyloxy-l-leucyl-l-leucyl-l-leucinal)–arrested HeLa Kyoto cells overexpressing mCherry or indicated mCherry-KIFBP construct. Scale bars, 2 μm. ACA, anti-centromere antibody; DAPI, 4′,6-diamidino-2-phenylindole. (**B**) Top: Graph of spindle lengths measured in cells overexpressing mCherry or indicated mCherry-KIFBP construct. Each dot represents a single cell. Means ± SD are displayed. Statistical results are shown for a one-way analysis of variance (ANOVA) with Tukey’s multiple comparisons test. n.s., not significant; **** indicates adjusted *P* < 0.0001 with 95% confidence interval. See fig. S13 for spindle length versus average mCherry expression for individual cells. Bottom: Graph of full width at half maximum (FWHM) of centromere fluorescence distribution along the length of the spindle measured in cells overexpressing mCherry of indicated mCherry-KIFBP construct. Each dot represents a single cell. Means ± SD are displayed. Statistical results are shown for a one-way ANOVA with Tukey’s multiple comparisons test. **** indicates adjusted *P* < 0.0001 with 95% confidence interval. See fig. S13 for FWHM distance versus average mCherry expression for individual cells. Data were obtained from a minimum of three independent experiments. The following cell numbers were analyzed for the mCherry and mCherry-KIFBP constructs: (i) mCherry (control) = 132 cells, (ii) mCherry-KIFBP-WT = 165 cells, (iii) mCherry-KIFBP-L1^m^ = 102 cells, (iv) mCherry-KIFBP-L14^m^ = 99 cells, and (v) mCherry-KIFBP-HP9b^m^ = 89 cells.

Overexpression of KIFBP in HeLa cells leads to defects in chromosome alignment and an increase in spindle length (*28*). To determine whether mutations that block KIFBP interaction in vitro also reduce KIFBP effects during mitosis, we transfected N-terminally mCherry-tagged KIFBP constructs into HeLa Kyoto cells and measured chromosome alignment and spindle length in metaphase-arrested cells. Consistent with previous results, overexpression of mCherry-KIFBP-WT decreased chromosome alignment, quantified by an increase in full width at half maximum (FWHM) of centromere fluorescence distribution along the length of the spindle (Fig. 7, A and B) (*28*, *39*). Overexpression of mCherry-KIFBP-WT also increased spindle lengths, as shown previously (*28*) (Fig. 7, A and B). These mitotic effects scaled with the mCherry-KIFBP-WT expression level, where cells expressing higher levels of mCherry-KIFBP-WT had longer spindles and more severe chromosome alignment defects (fig. S13).

To test the mitotic effects of the KIFBP mutants, we generated mCherry-KIFBP overexpression mutant constructs mCherry-KIFBP-L1^m^, mCherry-KIFBP-L14^m^, and mCherry-KIFBP-HP9b^m^ (Fig. 6B). Cells expressing mCherry-KIFBP-L1^m^ and mCherry-KIFBP-L14^m^ mutants displayed similar chromosome alignment and spindle lengths as cells overexpressing the mCherry control (Fig. 7, A and B). In contrast to mCherry-KIFBP-WT, mitotic effects of mCherry-KIFBP-L1^m^ did not scale with expression level, suggesting that mCherry-KIFBP-L1^m^ does not inhibit kinesin activity even when expressed at higher levels (fig. S13). Spindle length and chromosome alignment defects increased at high expression levels of mCherry-KIFBP-L14^m^, suggesting that mCherry-KIFBP-L14^m^ may inhibit kinesin activity at high expression levels (fig. S13). In contrast to mCherry-KIFBP-L1^m^ and mCherry-KIFBP-L14^m^, mCherry-KIFBP-HP9b^m^ showed similar effects as mCherry-KIFBP-WT overexpression, decreasing chromosome alignment and increasing spindle length (Fig. 7, A and B). Mitotic defects did not scale with expression level for mCherry-KIFBP-HP9b^m^, in contrast to mCherry-KIFBP-WT (fig. S13). Cells expressing lower levels of mCherry-KIFBP-HP9b^m^ displayed mitotic defects, suggesting that mCherry-KIFBP-HP9b^m^ may be a more potent inhibitor than mCherry-KIFBP-WT. These findings are consistent with the in vitro observations that KIFBP-L1^m^ and KIFBP-L14^m^ reduce KIFBP’s interaction with KIF15 and KIF18A, whereas the KIFBP-HP9b^m^ does not block interaction (Fig. 6, C to E).

**Fig. 8.**
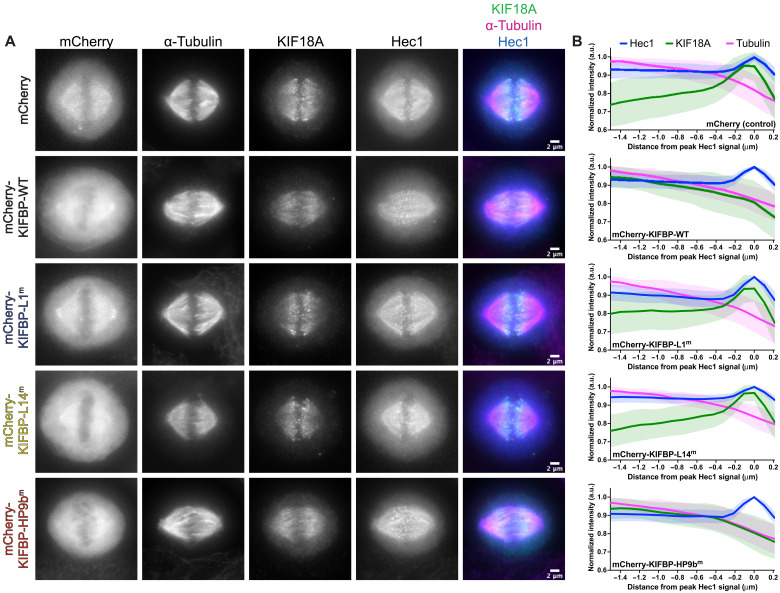
Mutations in KIFBP-L1 and KIFBP-L14 disrupt KIFBP regulation of KIF18A localization. (**A**) KIF18A localization in MG132-arrested HeLa Kyoto cells overexpressing mCherry or indicated mCherry-KIFBP construct. Hec1 is used as a marker for the kinetochore. Scale bars, 2 μm. (**B**) Line scan analyses of KIF18A distribution along kinetochore microtubules. Fluorescence values were normalized, aligned by peak Hec1 intensity, and averaged across multiple line scans. Hec1, blue; KIF18A, green; tubulin, magenta. Solid lines indicate the means, and shaded areas indicate SD. a.u., arbitrary units. The following cell numbers and line scans were analyzed for the mCherry and mCherry-KIFBP constructs: (i) mCherry (control) = 40 cells (64 lines), (ii) mCherry-KIFBP-WT = 34 cells (64 lines), (iii) mCherry-KIFBP-L1^m^ = 34 cells (64 lines), (iv) mCherry-KIFBP-L14^m^ = 32 cells (68 lines), and (v) mCherry-KIFBP-HP9b^m^ = 33 cells (63 lines).

To further investigate the cellular effects of the KIFBP mutations, we measured KIF18A localization in HeLa Kyoto cells overexpressing WT KIFBP or the KIFBP mutants. KIF18A accumulates at the plus ends of microtubules during metaphase, and we have previously shown that overexpression of mCherry-KIFBP-WT alters KIF18A spindle localization (*28*). Overexpression of mCherry-KIFBP-WT abolishes KIF18A plus-end enrichment and leads to a more uniform spindle localization (Fig. 8A), consistent with previous observations (*28*). Line scan analysis confirmed the loss of KIF18A from microtubule plus-end enrichment along individual kinetochore microtubules (Fig. 8B).

We predicted that mutations that abolished KIFBP interaction with kinesins in vitro would not disrupt KIF18A localization. Overexpression of mCherry-KIFBP-L1^m^ and mCherry-KIFBP-L14^m^ did not disrupt KIF18A plus-end enrichment on kinetochore microtubules (Fig. 8, A and B). In contrast, overexpression of mCherry-KIFBP-HP9b^m^ showed similar effects to overexpression of mCherry-KIFBP-WT (Fig. 8, A and B). This is especially interesting considering that KIFBP-L14^m^ binds KIF18A in vitro, suggesting that the interaction is not necessarily equivalent to inhibition. Even a 10% reduction in binding may be sufficient to impair regulation by KIFBP beyond a cellular threshold. Together, these results support the conclusion that KIFBP Loop-1 and Loop-14 are critical regions for kinesin interaction and indicate that both regions are necessary for KIFBP to limit KIF15 and KIF18A activity during mitosis.

### KIFBP-binding kinesins adopt conformations that are distinct from non–KIFBP-binding kinesins

To gain insight into how KIFBP inhibits particular kinesins, we performed a series of MD simulations using a number of kinesin family members. We selected two members that bind KIFBP (KIF15 and KIF18A) and two that show little or no interaction with KIFBP (KIF5C and KIF11) (*27*). Using only the motor domains, we performed 500+ nanosecond simulations of all-atom MD for each protein in the unbound, adenosine 5′-diphosphate (ADP) state (see Materials and Methods).

To analyze the dynamics and conformational spaces explored by these proteins, we compared MD trajectories between KIFBP-binding motors (KIF15 and KIF18A) and motors that do not bind KIFBP (KIF5C and KIF11). We performed principal components analysis (PCA) using the KIF15 simulation as the reference for the other proteins to reduce dimensionality. To minimize noise in the PCA that comes from fluctuations in unstructured regions of the protein, we only analyzed amino acids that were in stable secondary structure elements (ɑ helices of β strands) at least 80% of the time. Last, we removed ɑ4 from the PCA because this helix remained stably bound to the motors in the simulations (fig. S14) but is extended in the cryo-EM structure and would thus skew the PCA results.

**Fig. 9.**
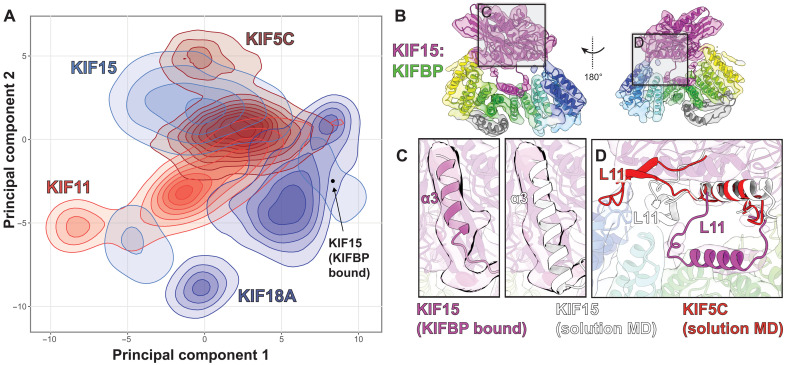
MD reveals specific conformations adopted by KIF15 and KIF18A that may promote KIFBP binding. (**A**) PCA of the MD simulations for all four kinesin motors (KIF15, KIF18A, KIF5C, and KIF11). The two KIFBP binders (KIF15 and KIF18A in shades of blue) can reach the conformation of the cryo-EM complex, but the nonbinders (KIF5C and KIF11 in shades of red) do not. (**B**) Overview of KIF15:KIFBP structure. (**C**) Comparison of KIF15-α3 between MD simulations and cryo-EM. (**D**) Differences in Loop-11 between KIF15 and KIF5C. For KIF15, Loop-11 fits within the KIFBP pocket, while the extended Loop-11 structure of KIF5C clashes with KIFBP.

Comparison of PCA for these kinesin motors shows that (i) all four proteins have similar ranges of motion and explore comparable regions of conformational space and (ii) there are specific motions that are only present in KIFBP-binding kinesins (KIF15 and KIF18A) (Fig. 9A). Close inspection of the amino acids that contribute to PC1 (i.e., the largest motions of KIF15) revealed that splaying of both the C-terminal end of ɑ3 and the N-terminal end of ɑ6 is the dominant contributor to this mode. These structural differences are consistent with the KIFBP:KIF15 cryo-EM structure (Fig. 2D) where movements of these helices are a defining feature of the bound complex. Thus, MD simulations indicate that only KIF15 and KIF18A are able to reach the conformation found in the bound complex (Fig. 9A).

The MD KIF15 solution structure differs in specific structural regions with KIFBP-bound KIF15. First, the KIF15 solution structure shows that KIF15-ɑ4 remains closely bound to the motor body throughout each simulation. This would suggest that the release and translocation of KIF15-ɑ4 would only occur upon interaction with KIFBP or on a much longer time scale than that sampled during the simulation. Second, although we saw that the movement of KIF15-ɑ3 was a key feature from the PCA, we see in the cryo-EM structure that the C-terminal end of KIF15-ɑ3 loses its structure over the final eight to nine residues, forming an extended loop with a short antiparallel β sheet (Fig. 9, B and C). This part of KIF15 makes contact with KIFBP, and it appears that this structural change would allow better contact between KIF15 and KIFBP.

Last, although ɑ4 has a nearly identical conformation for all kinesins, L11 is much more dynamic, and its conformation is kinesin dependent. For KIF5C, L11 tends to be extended away from ɑ4, and when superimposed on the cryo-EM complex, L11 has a steric clash with KIFBP (Fig. 9, B and D). Conversely, both KIF15 and KIF18A adopt more compact L11 structures, and these fit well within the cavity of the KIFBP structure (KIF15 structure shown in Fig. 9D). Together, this suggests that the overall conformation of the motor head and the dynamics or conformation of more flexible parts of each protein may act in concert to determine which kinesins will bind to KIFBP and which will not.

## DISCUSSION

Our work presents a previously undescribed mode of kinesin motor protein regulation via a multivalent interaction between KIFBP and the kinesin motor domain (movie S1). Using a combination of cryo-EM, XL-MS, MD simulations, biochemical assays, and cell biology, we describe a model in which KIFBP stabilizes the microtubule-binding ɑ4 helix (*5*, *40*–*42*) away from the kinesin motor domain in addition to sterically inhibiting the microtubule-binding interface. KIFBP does not mimic the negatively charged microtubule surface (*43*) to engage kinesin motors. Instead, KIFBP uses a hydrophobic cleft to hold ɑ4 and Loop-11 in a conformation that is incompatible with microtubule binding while simultaneously engaging and sterically inhibiting the kinesin microtubule-binding interface (Fig. 3).

Determining the binding mechanism of KIFBP relied on cryo-EM structural data for relatively small macromolecular samples. We determined a near-atomic structure of KIFBP corresponding to ~40 of 72 kDa, using this reconstruction for de novo model building. This places the KIFBP reconstruction and model among the smallest–molecular weight macromolecules to be built de novo by cryo-EM. The relatively small size of KIFBP may be the driving factor limiting the overall resolution of KIFBP alone to ~3.8 Å instead of obtaining higher-resolution reconstructions.

During the preparation of this work, another study used cryo-EM and cell biology assays to propose a mechanism of KIFBP-mediated kinesin inhibition (*34*). In this study, Atherton *et al.* (*34*) determined lower-resolution structures of KIFBP (4.8 Å) and KIFBP-KIF15 (6.9 Å) to arrive at a similar model of kinesin motor inhibition, where KIFBP stabilizes the ɑ4 helix away from KIF15. In our work, our higher-resolution structures of KIFBP alone (3.8 Å) and KIFBP-KIF15 (4.8 Å) allowed us to (i) build a high-confidence atomic model for KIFBP and (ii) map conformational changes in the KIF15 motor when bound to KIFBP. We showed that KIFBP uses a similar mode of inhibition for KIF18A, indicating that our proposed model is likely a general mode of kinesin inhibition. Last, we used our structures alongside XL-MS to map the interaction of KIFBP with kinesin motors and showed that blocking the interaction between KIFBP and KIF15 and KIF18A via mutagenesis minimizes its ability to regulate motor activity in the physiologically relevant context of mitosis (Figs. 7 and 8).

### Kinesin motor recognition by KIFBP

Our cryo-EM reconstruction of KIFBP reveals that KIFBP contains a 9-TPR array, which folds into a solenoid with a concave kinesin-interacting surface. Unlike continuous TPR proteins such as LGN (*44*), KIFBP is punctuated by a centrally located helix and loop (Fig. 1). The binding of KIFBP to kinesin motor heads is notably different from the interaction of many TPR proteins to their ligands (*35*). Many TPR proteins bind a short sequence, e.g., HOP binds the motif MEEVD in Hsp90 (*45*). In contrast, our cryo-EM and XL-MS show that the interaction of KIFBP with kinesin motors is highly multivalent. First, KIFBP-L1, localized at its N terminus, engages ɑ3 and β6 of the kinesin motor head. Second, KIFBP-L14 contacts β4-β5 of KIF15 and KIF18A with amino acid E168 in the KIF15 motor domain (or E161 in KIF18A) positioned to play a key role in this interaction. Third, ɑ4 helices of KIF15 and KIF18A become nestled into a multihelix groove created by KIFBP-HP4a, KIFBP-HP4b, and KIFBP-HP5a. The binding of KIF15, KIF18A, and KIF-HP-ɑ4 to KIFBP is notable because it requires a 15-Å displacement of the helix from its resting position within the kinesin motor head.

Our structure- and XL-MS–guided mutagenesis study of KIFBP revealed that KIFBP-L1 is especially important for motor binding. Charge neutralization of Loop-1 renders KIFBP incompetent for binding both KIF15 and KIF18A in vitro (Fig. 6). Consistent with these data, KIFBP-L1^m^ failed to produce phenotypes associated with KIFBP overexpression in cells, i.e., disruption of chromosome alignment and increased spindle length (Figs. 7 and 8). Unlike KIFBP-WT, the KIFBP-L1^m^ mutant was also incapable of disrupting KIF18A localization.

In contrast to KIFBP-L1, the role of KIFBP-L14 in motor inhibition remains less clear. Previous work using an artificial peroxisome transport assay suggested that KIFBP-L14 is important for the ability of KIFBP to inhibit the motor activities of KIF1A and KIF15 (*34*). However, mutation of KIFBP-L14 more strongly affected the ability of KIFBP to retrieve KIF1A from cell lysates than KIF15. In our experiments with purified proteins, we observed reduced binding of KIFBP-L14^m^ to KIF15 but only minimal reduction in binding to KIF18A (Fig. 6). Unexpectedly, in cells, KIFBP-L14^m^ reduced the ability of KIFBP to disrupt metaphase chromosome alignment, spindle length homeostasis, and KIF18A localization (Figs. 7 and 8). However, high levels of KIFBP-L14^m^ did produce phenotypes associated with KIFBP overexpression, suggesting that KIFBP-L14^m^ is capable of weak interactions with KIF18A and/or KIF15. This discrepancy in binding is unexpected because the region implicated in interaction with KIFBP-L14, kinesin Loop-8, is highly conserved between KIF15 and KIF18A. Furthermore, the precise binding site of KIFBP-L1 on KIF15/KIF18A is unclear, impeding a more detailed comparison between the KIFBP-L1 and KIFBP-L14 interaction surfaces. Together, these data suggest that KIFBP-L14 may be more important for binding some kinesins than others but that binding for all three kinesins collectively tested (KIF15, KIF18A, and KIF1A) was reduced beyond some cellular threshold necessary for producing the observed phenotypes of mitotic defects or reduced peroxisome transport. More work is required to relate the ability of KIFBP to bind kinesin motors in vitro to its ability to regulate kinesins in cells.

Last, we observed a high density of cross-links between KIFBP-HP9b (residues K610 and K617) and the microtubule-binding interface of KIF15 (Fig. 4). These structural elements are not within the cross-linking range of BS3 in our cryo-EM structures (Figs. 2 and 5), and the significance of these cross-links is therefore not clear. Perhaps in line with this, our analysis of the charge neutralization mutant KIFBP-HP9b^m^ revealed that this mutation had little effect on in vitro interaction with KIF15 or KIF18A or on the mitotic phenotypes that we quantified, suggesting that electrostatic interactions with amino acids 610 to 617 of KIFBP are not critical for kinesin interaction. A role for the C terminus in KIFBP-motor interactions should not be dismissed, as a recent study identified a novel nonsense KIFBP mutation in a patient with GOSHS that truncates the protein at position 593 (*46*). It will be interesting to determine whether the C terminus of KIFBP is generally important for its interaction with all kinesins or whether it instead drives interactions with kinesins that are more clinically relevant to GOSHS.

### KIFBP remodels the kinesin motor head to displace kinesin α4

Kinesin ɑ4 helix plays a critical role in motor-KIFBP binding. Lysine residues within KIFBP-HP4a (K205) and KIFBP-LH (K307) cross-link residues located in KIF15-L11 (K273 and K283). Unexpectedly, residues in KIFBP-L1 (K26, K30, and K36) also cross-linked KIF15 residues K273 and K283. The significance of these cross-links is not clear, but these data may suggest that an intermediary complex between KIFBP and kinesin motor domains, driven by the interaction of KIFBP-L1 with kinesin Loop11, may form before the acquisition of the final bound state.

One outstanding question concerns the mechanism by which KIF15/KIF18A ɑ4 undergoes long-range motion to achieve KIFBP binding. The simplest possibility is that ɑ4 is positionally unstable. If sufficiently compliant, then the adjacent loops, i.e., Loop-11 and Loop-12, may allow ɑ4 excursions that eventually result in “capture” of ɑ4 by KIFBP. Long-range motions of ɑ4 are not without precedent. For example, Wang *et al.* (*47*) observed by x-ray crystallography that ɑ4 of KIF19 is positioned much farther from the motor head than is typical. Our MD work, however, suggests that ɑ4 remains closely associated with the motor head (fig. S14), leading us to speculate that the binding of a motor head by KIFBP results in allosteric changes in the structure of both proteins, inducing motions of ɑ4 that predispose it to KIFBP binding. Comparison of KIF15 in apo versus bound states supports this possibility (Fig. 2, D and E). When bound to KIFBP, KIF15 showed a shift of several α helices (α1, α3, and α6) away from the core of the motor as well as large movements of several β-strand pairs. The movement of these structural elements causes the motor to assume a more open conformation.

Another unresolved issue is the functional relevance of ɑ4 extraction by KIFBP. ɑ4 is displaced a substantial distance from its position in the motor head (Fig. 2, A to C), but it is unclear why this displacement is advantageous for the mechanism of action by KIFBP. In principle, steric inhibition of the KIF15/KIF18A microtubule-binding domain should be sufficient to prevent motor-microtubule binding. One possibility is that ɑ4 extraction is necessary for the formation of a stable, long-lived complex; however, as mentioned above, our MD analysis suggests that ɑ4 is only available for extraction due to allosteric changes caused by KIFBP binding. In addition, mutating KIFBP Loop-1 disables both in vitro interaction (Fig. 6) and motor inhibition in cells (Figs. 7 and 8), indicating that ɑ4 displacement is not sufficient for complex formation. Another possibility is that ɑ4 extraction is necessary for complex maintenance, perhaps serving to lock the motor in a bound conformation. It is also yet unknown how KIFBP dissociates from kinesin motor domains; it is possible that the interaction of ɑ4 with HP4a/b-HP5 may be stable enough to require some form of active regulation for disengagement. Future work is required to elucidate the functional implications of ɑ4 extraction.

In summary, our work establishes a structural mechanism by which KIFBP inactivates the microtubule-binding activity of mitotic kinesins KIF15 and KIF18A. Unlike common TPR tandem proteins, KIFBP uses multivalency to form a complex with the kinesin motor head. Multivalency may explain why it has not been possible to identify a consensus sequence for kinesin motors that bind KIFBP versus those that do not (*34*). Our MD simulations and PCA also indicate that motor-specific steric clashes may serve as a mechanism that prevents certain motors from binding KIFBP. Specifically, we observed that L11 of KIF5C would sterically clash with KIFBP-HP2, whereas L11 of KIF15 and KIF18A fits within the cavity between HP3 and HP4 of KIFBP. Further work is required to test the generality of this idea. An additional area for future work is to reveal the mechanism by which KIFBP dissociates from a kinesin motor. The multivalency with which KIFBP interacts with a kinesin motor, in particular the interaction of HP4a/b-HP5 with ɑ4, suggests that a motor will not readily disengage from KIFBP. Motor recycling may require active regulation, e.g., phosphorylation, as proposed in earlier work (*27*).

## MATERIALS AND METHODS

### Plasmid construction

The following plasmids that were used in this study were previously described elsewhere: GST-KIFBP (*28*), mCherry, and mCherry-KIFBP expression plasmids (*28*). The construction of the other plasmids used in this study is described as follows.

His_6_-KIF15-N375 was created through isothermal assembly where the first 375 amino acids of the KIF15 open reading frame were amplified from pEGFP-C1-Kif15-FL (*48*) and inserted into the pET15b vector. Correct insertion was confirmed by sequencing.

His_6_-KIF18A-N363 was created through isothermal assembly where a gBlock gene fragment of the first 363 amino acids of KIF18A codon-optimized for expression in *Escherichia coli* (IDT) was inserted into the pET15b vector. Correct insertion was confirmed by sequencing.

GST-KIFBP-L1^m^ was created by site-directed mutagenesis of GST-KIFBP, replacing amino acids 21 to 40 with the altered amino acid sequence described in Fig. 6B. Similarly, GST-KIFBP-HP9b^m^ was created by site-directed mutagenesis of GST-KIFBP, replacing amino acids 610 to 617 with the altered amino acid sequence described in Fig. 6B. Mutagenesis was confirmed by sequencing of the open reading frame. mCherry-KIFBP-L1^m^ and mCherry-KIFBP-HP9b^m^ were created in the same manner by site-directed mutagenesis of the mCherry-KIFBP WT plasmid. To create the mCherry-KIFBP-L14^m^ plasmid, a GeneStrand containing KIFBP base pairs 1092 to 1588 with the L14^m^ mutations was synthesized (Eurofins). This gene fragment was then inserted into the mCherry-KIFBP expression vector by isothermal assembly using the commercially available Gibson Assembly Master Mix (New England Biolabs) after PCR amplification of the mCherry-KIFBP expression vector.

### Protein expression and purification

Expression of GST-KIFBP, GST-KIFBP-L1^m^, GST-KIFBP-L14^m^, and GST-KIFBP-HP9b^m^ was induced in BL21-DE3 cells with 0.4 M isopropyl-β-d-thiogalactopyranoside (IPTG) overnight at 16°C. Cells were pelleted and resuspended in lysis buffer {1× phosphate-buffered saline (PBS), 0.5 mM NaCl, 5 mM β-mercaptoethanol, 1% NP-40, and protease inhibitors [1 mM phenylmethylsulfonyl fluoride (PMSF), 1 mM benzamidine, and lysophosphatidylcholine (LPC; 10 μg/ml)]}, after which they were incubated with lysozyme (1 mg/ml) for 30 min on ice followed by sonication. The lysate was clarified by centrifugation for 30 min at 35,000 rpm at 4°C in a Type 45 Ti rotor (Beckman). Cleared lysate was incubated with 2 ml of glutathione-Sepharose (Thermo Fisher Scientific) for 1 hour and washed with 50 ml [25 CV (column volume)] of wash buffer (1× PBS, 0.5 M NaCl, and 5 mM β-mercaptoethanol). Resin was incubated with 200 μl of PreScission Protease (Cytiva) in 2 ml of cleavage buffer [50 mM tris-HCl (pH 7.0), 150 mM NaCl, 1 mM EDTA, and 1 mM dithiothreitol (DTT)] for 4 hours at 4°C to cleave the GST tag. Protein was eluted with 50 mM tris-HCl (pH 8.0), and peak fractions were combined and clarified by centrifugation for 5 min at 20,000 rpm at 4°C, after which they were subjected to size exclusion chromatography on a Superdex 200 column (GE Healthcare) equilibrated in 10 mM K-Hepes (pH 7.7), 50 mM KCl, and 1 mM DTT. Protein concentration of fractions after gel filtration was estimated using a Bradford assay, after which peak fractions were combined, concentrated to >1 mg/ml using Amicon 10-kDa centrifugal filter units (Millipore), and either used immediately for cryo-EM or flash-frozen and stored at −80°C.

Expression of His_6_-KIF15-N375 and His_6_-KIF18A-N363 was induced in BL21-DE3 cells with 0.4 M IPTG overnight at 16°C. Cells were pelleted and resuspended in lysis buffer {1× PNI (50 mM sodium phosphate, 500 mM NaCl, and 20 mM imidazole), 1% NP-40, 1 mM MgATP, and protease inhibitors [1 mM PMSF, 1 mM benzamidine, and LPC (10 μg/ml)]}, after which they were incubated with lysozyme (1 mg/ml) for 30 min on ice followed by sonication. The lysate was clarified by centrifugation for 30 min at 35,000 rpm at 4°C in a Type 45 Ti rotor (Beckman). Cleared lysate was incubated with 2 ml of Ni-NTA (nitrilotriacetic acid) agarose (Qiagen) for 1 hour and washed with 50 ml of wash buffer (1× PNI, 100 μM MgATP, and 5 mM β-mercaptoethanol). Protein was eluted with elution buffer (1× PNI, 100 μM MgATP, 5 mM β-mercaptoethanol, and 200 mM imidazole), and peak fractions were combined and clarified by centrifugation for 5 min at 20,000 rpm at 4°C, after which they were subjected to size exclusion chromatography on a Superdex 200 column equilibrated in gel filtration buffer [10 mM K-Hepes (pH 7.7), 50 mM KCl, 1 mM DTT, and 0.2 mM MgATP]. Protein concentration of fractions after gel filtration was estimated using a Bradford assay, after which peak fractions were combined and concentrated to >1 mg/ml using Amicon 10-kDa centrifugal filter units (Millipore). Before cryo-EM grid preparation, the protein was then mixed with equimolar KIFBP and subjected to size exclusion chromatography for a second time on a Superose 6 column (GE Healthcare) equilibrated with gel filtration buffer. Peak fractions were analyzed by SDS-PAGE and stained with Coomassie blue. Fractions containing only the two proteins of interest were then combined, concentrated to >1 mg/ml, and used immediately for cryo-EM.

### Cryo-EM grid preparation and data collection

For cryo-EM grid preparation, after size exclusion chromatography, KIFBP was concentrated to 4 mg/ml, whereas KIFBP:KIF15 and KIFBP:KIF18A complexes were each concentrated to 1 mg/ml. Aliquots of 4 μl were applied on glow-discharged UltrAuFoil R(1.2/1.3) 300-mesh gold grids (Electron Microscopy Sciences). The grids were then blotted with filter paper and plunge-frozen into liquid ethane cooled by liquid nitrogen using a Vitrobot Mark IV (Thermo Fisher Scientific) set to 4°C, 100% humidity, 1.5-s blot, and a force of 20.

For KIFBP and KIFBP:KIF15 samples, datasets were collected using Leginon (*49*) on a Thermo Fisher Scientific Glacios transmission electron microscope operating at 200 keV equipped with a Gatan K2 Summit direct electron detector (Gatan Inc.) in counting mode. For KIFBP, a total of 11,086 micrographs were collected through three data collection sessions with total doses of 58 to 68 e^−^/Å^2^ during an exposure time of 7 to 9 s, dose fractionated into 35 to 45 movie frames at defocus ranges of 1 to 2 μm. The magnification used here is ×45,000, resulting in a physical pixel size of 0.98 Å per pixel. For KIFBP:KIF15, four data collection sessions were performed with ×45,000 magnification and a physical pixel size of 0.98 Å per pixel. A total number of 6184 micrographs were collected with a total dose of 60 e^−^/Å^2^ during 6 to 7 s of exposure time, dose fractionated into 30 to 35 movie frames.

Data collection for the KIFBP:KIF18A sample was automatically collected using Leginon (*49*) on an FEI Talos Arctica transmission electron microscope operating at 200 keV equipped with a Gatan K2 Summit direct electron detector in counting mode. Three datasets were collected, resulting in a total of 4669 micrographs in a physical pixel size of 0.91 Å per pixel. The total dose ranges from 52 to 62 e^−^/Å^2^ in 7 to 8 s of exposure time with dose fractionated into 35 to 40 movie frames.

### Cryo-EM data processing

The data processing diagram for KIFBP is shown in figs. S2 and S3. Movie alignment, Contrast Transfer Function (CTF) parameter estimation, and particle picking were performed using Warp (*50*). The resulting particles were imported into cryoSPARC (*51*) and underwent iterative 2D classification to remove incorrect particle picks. We initially analyzed sample heterogeneity in “dataset3” using ab initio reconstruction with three classes. Particles from the one higher-resolution class were then subjected to another round of ab initio reconstruction with two classes, resulting in two very similar classes. After careful examination, we believe that the first class is the full-length KIFBP map, while the other is the KIFBP lack of the C-terminal helices, explaining why some class averages are missing the C-terminal helix pairs of the KIFBP.

To resolve the structure of the full KIFBP, we deliberately selected the class averages from “dataset1” and “dataset2” that resemble the full KIFBP molecule using cryoSPARC (*51*). These particles were then combined with the particles from the full-length KIFBP class in dataset3 and subjected to ab initio reconstruction with two classes. Particles from the higher-resolution classes were selected for nonuniform refinement (*52*) to obtain a 4.7-Å-resolution map. The map quality was improved to 4.6 Å using local refinement with a static mask. The resulting map was manually sharpened for the visual inspection purpose using a B-factor of −50 Å^2^.

Next, we focused on analyzing the N terminus of KIFBP lacking the C-terminal helices to try to improve the resolution. Particles from dataset2 were chosen because CTF fit resolution in dataset2 was the best among all three datasets. A total of 913,455 particles from Warp (*50*) were imported into cryoSPARC (*51*). We then performed iterative heterogeneous refinement with one class from the KIFBP lacking the C-terminal helices and two low-resolution classes from failed ab initio reconstruction jobs. A total of 301,491 particles corresponding to KIFBP were enriched after extensive heterogeneous refinement and 2D classification. These particles were then subjected to homogeneous refinement and local refinement, resulting in a 3.8-Å-resolution map. However, the quality of the map was not satisfactory. To improve the map quality, the particles were exported into RELION-3.1 (*53*) and underwent one round of 3D autorefinement to obtain a reconstruction at 4.2 Å. Subsequently, two rounds of CTF refinement were performed to correct for beam tilt (*54*). 3D autorefinement with refined beam tilt yielded an estimated resolution of 4.1 Å. We then exported micrograph motion trajectories from Warp and performed Bayesian polishing (*55*) to optimize per-particle motion tracks. 3D autorefinement from the polished particles resulted in a 3.8-Å map. Following this step, one round of 3D classification with six classes was performed to further remove heterogeneity. Five classes corresponding to KIFBP lacking the C terminus were selected and underwent another round of refinement, yielding a 3.8-Å-resolution map. The particles were then reextracted and recentered. The following 3D autorefinement yielded a 4-Å map. Bayesian polishing was performed on this particle stack, resulting in an improved map quality. 3D classification without alignment using *T* = 12 resulted in one high-resolution class. 3D autorefinement using the 128,190 particles from this high-resolution class gave a 3.8-Å map with improved map quality.

For KIFBP:KIF15, all the data processing steps were performed in cryoSPARC (*51*), as presented in fig. S8. To generate an initial map for the KIFBP:KIF15 complex, 1007 movies from dataset1 were imported into cryoSAPRC (*51*). Patch motion correction and patch CTF estimation were used to correct beam-induced motion and estimate CTF parameters. A total of 235,721 particles were automatically picked using the Topaz general model (*56*). These particles were then subjected to iterative 2D classification to remove incorrect particle picks. The resulting 32,262 particles were used for ab initio reconstruction with one class and also retraining Topaz. The model from the ab initio reconstruction was refined to ~7 Å and used as a template for the heterogeneous refinement in the following steps.

Movies from dataset1, dataset2, dataset3, and dataset4 were imported into cryoSPARC (*51*) and processed separately at the beginning steps. Movies were aligned using patch motion correction with dose weighting. CTF parameters were estimated with patch CTF estimation. Micrographs with CTF fit resolution below 5 Å were selected and subjected to particle picking using a restrained Topaz model. The picked particles underwent one round of 2D classification to remove incorrect particle picks. We then performed iterative heterogeneous refinement with one class from the initial template and two low-resolution classes from the early terminated ab initio reconstruction jobs to enrich particles corresponding to KIFBP:KIF15 complex. The resulting particles were further cleaned by 2D classification and ab initio reconstruction with multiclasses. Particles from the individual dataset were then reextracted, recentered, and combined, resulting in 189,984 particles. These particles were subsequently classified into three classes using ab initio reconstruction. Two classes showing KIFBP and KIF15 density were merged and further classified into two classes with ab initio reconstruction. One class with the better KIF15 motor domain density was selected and subjected to homogeneous refinement, resulting in a 4.8-Å-resolution map. Then, local refinement with a user-defined mask was performed to improve the map quality.

For KIFBP:KIF18A, the data processing diagram is presented in fig. S11. A total 4669 micrographs were collected through three datasets. For each dataset, motion correction, CTF estimation, and particle picking were performed in Warp (*50*), resulting in 71,529 particles (dataset1), 94,716 particles (dataset2), and 638,186 particles (dataset3). These particles were imported into cryoSPARC (*51*) and underwent interactive 2D classification to remove incorrect particle picks. The remaining 156,159 particles were used for ab initio reconstruction into four classes in cryoSPARC. A total of 54,801 particles from the class with clear KIF18A density were selected for homogeneous refinement to obtain a 5-Å-resolution structure of the KIFBP:KIF18A complex. The quality of the map was further improved by local refinement in cryoSPARC with a user-defined mask.

### Model building

To construct an atomic model of KIFBP, first, we began by de novo building of the 3.8-Å KIFBP reconstruction using Coot (*57*) on the RELION (*58*) postprocessed reconstruction. To guide model building, we used density modification with DeepEMhancer (*59*) that was run on the COSMIC^2^ science gateway (*60*) to help interpret the cryo-EM density. From this process, we built amino acids 5 to 403 of KIFBP, and the RELION postprocessed map was used for model refinement and validation using Phenix (*61*). After building this high-resolution part of our reconstruction, we built polyalanine models for the C-terminal helices KIFBP-HP6b, KIFBP-HP7, KIFBP-HP8, and KIFBP-HP9 using Coot (*59*). The manual build model was then subjected to real-space refinement in Phenix (*61*).

Because of the moderate resolution (4.8 Å) of KIF15:KIFBP, we built the model of KIF15:KIFBP using a combination of Rosetta-CM (*36*), Rosetta-Relax, and manual building in Coot (*57*). For the KIFBP model, we manually docked the KIFBP model into the density using Chimera (*62*), after which we fit the model into the density Rosetta-Relax. To fit KIF15 into the density, we manually docked KIF15 [Protein Data Bank (PDB): 4BN2] (*37*) into the cryo-EM density, removing KIF15-L11, KIF15-α4, and KIF15-L12 from the model. With this docking, we then ran Rosetta-CM (*36*), using atomic models 1V8K (Chain A) (*63*), 2OWM (Chain B) (*64*), 3U06 (Chain A) (*65*), 4BN2 (Chain C) (*37*), 5GSZ (Chain A) (*66*), 5MIO (Chain C) (*67*), 5MLV (Chain D) (*68*), 5MM4 (Chain K) (*69*), 5MM7 (Chain K) (*69*), and 6B0I (Chain K) (*70*) as the library of fragments for rebuilding. After running Rosetta-CM to calculate 5000 models, we used the lowest scoring model for the final step of Rosetta-Relax. To build KIF15-L11, KIF15-α4, and KIF15-L12, we built a polyalanine model manually using Coot (*57*).

For the KIF18A:KIFBP model, we used Rosetta-Relax to fit the KIFBP model into the density. For the KIF18A motor, we manually docked the crystal structure of KIF18A (PDB: 3LRE) (*71*) into the density with the exception of KIF18A-L11, KIF18A-α4, and KIF18A-L12. To build KIF18A-L11, KIF18A-α4, and KIF18A-L12, we built a polyalanine model manually using Coot (*57*).

The efficiency (cryoEF) (*72*) for each reconstruction was calculated on the COSMIC^2^ science gateway (*60*). Figures were prepared using Chimera (*62*) and Chimera X (*73*).

### Cross-linking mass spectrometry

His_6_-KIF15-N375 and KIFBP were purified as described above. An equimolar solution of both proteins was prepared in a cross-linking buffer [40 mM Hepes (pH 7.4)] where the total protein concentration was 10 μM and the amount of each protein was at least 20 μg. A 50 mM solution of the 11-Å lysine-targeting cross-linker BS3 was prepared in water and added to the reaction in a 100 M excess. The reaction proceeded for 30 min while rotating at 4°C, after which it was quenched with tris-HCl (pH 7.5) at a final concentration of 50 mM. As an uncross-linked control, a separate reaction was prepared and quenched the same way, but no cross-linker was added.

The cross-linking reactions were resuspended in 50 μl of 0.1 M ammonium bicarbonate buffer (pH ~8). Cysteines were reduced by adding 50 μl of 10 mM DTT and incubating at 45°C for 30 min. Samples were cooled to room temperature, and alkylation of cysteines was achieved by incubating with 65 mM 2-chloroacetamide, under darkness, for 30 min at room temperature. Overnight digestion with 1:50 enzyme:substrate modified trypsin was carried out at 37°C with constant shaking in a ThermoMixer. Digestion was stopped by acidification, and peptides were desalted using SepPak C18 cartridges using the manufacturer’s protocol (Waters). Samples were completely dried via Vacufuge. Resulting peptides were dissolved in 9 μl of 0.1% formic acid/2% acetonitrile solution, and 2 μl of the peptide solution was resolved on a nano-capillary reverse-phase column (Acclaim PepMap C18, 2 μm, 50 cm, Thermo Fisher Scientific) using a 0.1% formic acid/2% acetonitrile (buffer A) and 0.1% formic acid/95% acetonitrile (buffer B) gradient at 300 nl/min over a period of 180 min (2 to 25% buffer B in 110 min, 25 to 40% in 20 min, and 40 to 90% in 5 min followed by holding at 90% buffer B for 10 min and equilibration with buffer A for 30 min). The eluent was directly introduced into a Q Exactive HF mass spectrometer (Thermo Fisher Scientific, San Jose, CA) using an EasySpray source. MS1 scans were acquired at 60 K resolution [automatic gain control (AGC) target, 3 × 10^6^; max Injection time (IT), 50 ms]. Data-dependent collision-induced dissociation MS/MS spectra were acquired using the Top speed method (3 s) following each MS1 scan (normalized collision energy, ~28%; 15 K resolution; AGC target, 1 × 10^5^; max IT, 45 ms).

pLink v2.3.9 was used to perform database searching against a FASTA protein sequence file containing full-length KIF15, KIFBP, and 292 common contaminant proteins. Raw data files were searched with BS3 as the cross-linker, Trypsin_P allowing up to three missed cleavages, peptide mass between 500 and 6000, peptide length between 5 and 60, precursor and fragment tolerances set to 20 parts per million (ppm), fixed carbamidomethyl cysteine, variable methionine oxidation, 10-ppm filter tolerance, and separate 5% peptide spectrum match (PSM) false discovery rate (FDR). pLabel v2.4.1 was used to visualize cross-linked MS/MS spectra. Of the resulting FDR-filtered list of cross-linked peptides, we filtered out all intra-KIFBP and intra-KIF15 cross-links, as well as cross-links with contaminant proteins and cross-links with an *e* value of >0.05.

### In vitro pull-down assays

One hundred microliters of Ni-NTA agarose resin (Qiagen) per condition was equilibrated with binding buffer [10 mM Hepes (pH 7.4), 50 mM KCl, and 10 mM imidazole] and incubated with 250 μg of either His_6_-KIF15-N375 or His_6_-KIF18A-N363 for 30 min at 4°C. The kinesin-bound resin was then washed three times in batch with 10 CV of binding buffer, split into 20-μl aliquots, and incubated with 20 μg of either KIFBP-WT, KIFBP-L1^m^, KIFBP-L14^m^, KIFBP-HP9b^m^, or GST in a total volume of 200 μl for 30 min at 4°C. To control for nonspecific binding to the resin, 20 μl of resin was incubated with either 20 μg of WT-KIFBP or GST with no previous kinesin incubation step. After incubation with GST or KIFBP proteins, the resin was pelleted, and the supernatant was removed and saved for analysis. The resin was then washed five times with 10 CV of wash buffer (binding buffer with 0.05% Tween 20). After the final wash, each resin sample was resuspended in 80 μl of binding buffer, and samples were taken for analysis. Five percent of each supernatant and pellet sample was boiled in 5× SDS-sample dye and loaded onto a 10% SDS-PAGE gel. Gels were stained with Coomassie blue, and quantifications of band intensities were done with ImageJ. Images for publication were enhanced with ImageJ, while quantification was done from raw images.

### Cell culture and transfections

HeLa Kyoto cells were cultured at 37°C with 5% CO_2_ in MEM-α medium (Gibco) containing 10% fetal bovine serum (Gibco). For plasmid transfections in a 24-well plate format, ~75,000 cells in 500 μl of MEM-α medium were seeded onto acid-washed glass coverslips and subsequently transfected with 375 ng of mCherry alone or mCherry-KIFBP plasmid DNA (containing WT KIFBP sequence or indicated KIFBP mutant). Cells were treated with mCherry and indicated mCherry-KIFBP plasmids that were preincubated for 10 min in 50 μl of Opti-MEM (Gibco) and 1 μl of Lipofectamine LTX reagent (Invitrogen). Plasmid transfections were incubated for 24 hours before fixation for immunofluorescence.

### Cell fixation and immunofluorescence

For metaphase observations of spindle length and chromosome alignment, cells expressing mCherry and mCherry-KIFBP (WT or indicated mutants) were treated with 20 μM MG132 (*N*-carbobenzyloxy-l-leucyl-l-leucyl-l-leucinal) (Selleck Chemicals) for 2 hours before fixation. Cells were fixed on coverslips in −20°C methanol (Thermo Fisher Scientific) with 1% paraformaldehyde (Electron Microscopy Sciences) for 10 min on ice. Coverslips were then washed three times for 5 min each in tris-buffered saline [TBS; 150 mM NaCl and 50 mM tris base (pH 7.4)]. Coverslips were blocked for 1 hour at room temperature in 20% goat serum in antibody dilution buffer [Abdil: TBS (pH 7.4), 1% bovine serum albumin, 0.1% Triton X-100, and 0.1% sodium azide]. Coverslips were then washed two times in TBS for 5 min each before the addition of primary antibodies. Primary antibodies were diluted in Abdil. For KIF18A localization analyses, the following primary antibodies were used at the indicated dilutions: rat anti–α-tubulin 1:500 (MAB1864; Sigma-Aldrich), rabbit anti-KIF18A 1:100 (A301-080A; Bethyl), and mouse anti-Hec1 1:500 (GTX70268; GeneTex). All mCherry images for KIF18A localization analyses are direct mCherry fluorescence. For KIF18A localization analyses, the following secondary antibodies were used at 1:500 dilution: goat anti-rabbit immunoglobulin G (IgG) conjugated to Alexa Fluor 488 (A11034; Invitrogen), goat anti-mouse IgG conjugated to Alexa Fluor 405 (A31553; Invitrogen), and goat anti-rat IgG conjugated to Alexa Fluor 647 (A21247; Invitrogen). For spindle length and chromosome alignment analyses, the following primary antibodies were used at the indicated dilutions: mouse anti–γ-tubulin 1:500 (T5326; Sigma-Aldrich), rabbit anti-mCherry 1:500 (ab167453; Abcam), and human anti-centromere antibody (ACA) 1:250 (15-235; Antibodies Inc.). All primary antibodies were incubated for 1 hour at room temperature with the exception of the human ACA antibody, which was incubated at 4°C overnight. For spindle length and chromosome alignment analyses, the following secondary antibodies were used at 1:500 dilution: goat anti-human IgG conjugated to Alexa Fluor 488 (A11013; Invitrogen), goat anti-mouse IgG conjugated to Alexa Fluor 647 (A21236; Invitrogen), and goat anti-rabbit IgG conjugated to Alexa Fluor 594 (A11037; Invitrogen). Coverslips were washed two times in TBS for 5 min each between primary and secondary antibody incubations. Coverslips were washed three times in TBS for 5 min each before mounting coverslips with ProLong Gold Antifade mounting medium with 4′,6-diamidino-2-phenylindole (DAPI) (spindle length and chromosome alignment analyses) (P36935, Invitrogen) or ProLong Gold Antifade mounting medium without DAPI (KIF18A localization analyses) (P36934, Invitrogen). Coverslips were imaged on a Ti-E inverted microscope (Nikon Instruments) using a Plan Apo λ 60× 1.42 numerical aperture objective, environmental chamber at 37°C, a Clara cooled charge-coupled device camera (Andor), and Nikon Elements Software (Nikon Instruments).

### Chromosome alignment analysis

Cells expressing mCherry or indicated mCherry-KIFBP constructs were fixed and stained for mCherry, γ-tubulin, and ACA as described above. As described previously (*28*, *39*), single focal plane images with both spindle poles in focus were acquired. A boxed region of interest with a fixed height and width defined by the length of the spindle was used to measure the distribution of ACA-labeled kinetochore fluorescence using the Plot Profile command in Fiji. The ACA signal intensity was normalized internally to its highest value and plotted as a function of distance along the pole-to-pole axis. These plots were then fitted to a Gaussian curve, and the FWHM for the Gaussian fit and the spindle length are reported for each cell analyzed. Means and SDs are reported from a minimum of three independent experiments for each construct. The following cell numbers were analyzed for the indicated mCherry and mCherry-KIFBP constructs: (i) mCherry (control) = 132 cells, (ii) mCherry-KIFBP-WT = 165 cells, (iii) mCherry-KIFBP-L1^m^ = 102 cells, (iv) mCherry-KIFBP-L14^m^ = 99 cells, and (v) mCherry-KIFBP-HP9b^m^ = 89 cells.

### KIF18A line scan analysis

Cells expressing mCherry or indicated mCherry-KIFBP constructs were fixed and stained for endogenous KIF18A, α-tubulin, and Hec1 as described above. Cells were imaged with 0.2-μm z-stacks throughout the entire cell. Within these z-sections, 2-μm line scans were manually drawn in Fiji for individual kinetochore microtubules (one to three line scans per cell), and the profile intensities along those lines were measured and recorded for the KIF18A, α-tubulin, and Hec1 channels. Each of these profile intensities for KIF18A, α-tubulin, and Hec1 was normalized internally to its highest value. These normalized line scans were then aligned by peak Hec1 intensity and averaged for each pixel distance. Means and SDs are reported from a minimum of three independent experiments for each construct. The following cell numbers and line scans were analyzed for the indicated mCherry and mCherry-KIFBP constructs: (i) mCherry (control) = 40 cells (64 lines), (ii) mCherry-KIFBP-WT = 34 cells (64 lines), (iii) mCherry-KIFBP-L1^m^ = 34 cells (64 lines), (iv) mCherry-KIFBP-L14^m^ = 32 cells (68 lines), and (v) mCherry-KIFBP-HP9b^m^ = 33 cells (63 lines).

### MD simulations and analysis

The structures of KIF5C, KIF15, KIF18A, and KIF11 bound to ADP and Mg^2+^ were taken from PDB structures 1BG2 (*74*), 4BN2 (*37*), 3LRE (*71*), and 1II6 (*75*), respectively. The missing residues of KIF5C were filled in as previously described (*76*). For all other proteins, I-TASSER was used to fill in the gaps of the remaining structures using the PDBs as primary template (*77*–*79*). AmberTools was then used to prepare all systems for simulation (*80*). Each system was solvated with a box of TIP3P water molecules with 10-Å padding around the protein. Na^+^ and Cl^−^ were added to both neutralize the systems and set the ionic concentration to 50 mM. NAMD was used to carry out the MD simulations with the amber ff19SB force field (*81*, *82*). Force field parameters for the ADP nucleotide were obtained from the AMBER parameter database (*83*). Following minimization, heating, and equilibration, the systems were simulated at 300 K and 1 atm of pressure in an NpT ensemble. To allow for 2-fs time steps, bonded hydrogens were fixed. For long-range electrostatics, particle mesh Ewald was used with a 10-Å cutoff and 8.5-Å switch distance for van der Waals interactions (*84*). MD simulations were completed in 100-ns replicates starting from random velocities for a total simulation time of 500 ns for KIF5C, KIF11, and KIF18A and 600 ns for KIF15.

All analysis was carried out using the Bio3d package (v2.4.1) in R (*85*). We first aligned our four kinesins of interest and then restricted analysis to the amino acids that appeared in all the motors. To focus on large-scale rearrangements in the motor head and remove the noise from fluctuating loops, we next restricted analysis to amino acids in stable secondary structures, leaving us with 154 amino acid positions in each protein. We performed PCA using KIF15 as the reference structure. The remaining three kinesin simulations were then projected onto this KIF15 PCA space for direct comparison with each other and the cryo-EM structure.

## Notes

View/request a protocol for this paper from *Bio-protocol*.
