# Supplementary material for: Kinesin-binding protein remodels the kinesin motor to prevent microtubule binding
Source: Sci Adv. 2021 Nov 19;7(47):eabj9812. doi: 10.1126/sciadv.abj9812 (PMC8604404; doi:10.1126/sciadv.abj9812)
Supplement: Supplementary file 1 — Figs. S1 to S14 Tables S1 to S5 Legend for movie S1 [file sciadv.abj9812_sm.pdf]

Science Advances
AAAS

Supplementary Materials for

Kinesin-binding protein remodels the kinesin motor to prevent microtubule binding

April L. Solon, Zhenyu Tan, Katherine L. Schutt, Lauren Jepsen, Sarah E. Haynes, Alexey I. Nesvizhskii, David Sept, Jason Stumpff, Ryoma Ohi*, Michael A. Cianfrocco*

*Corresponding author. Email: oryoma@umich.edu (R.O.); mcianfro@umich.edu (M.A.C.)

Published 19 November 2021, *Sci. Adv.* **7**, eabj9812 (2021)
DOI: 10.1126/sciadv.abj9812

The PDF file includes:

Figs. S1 to S14
Tables S1 to S5
Legend for movie S1

Other Supplementary Material for this manuscript includes the following:

Movie S1

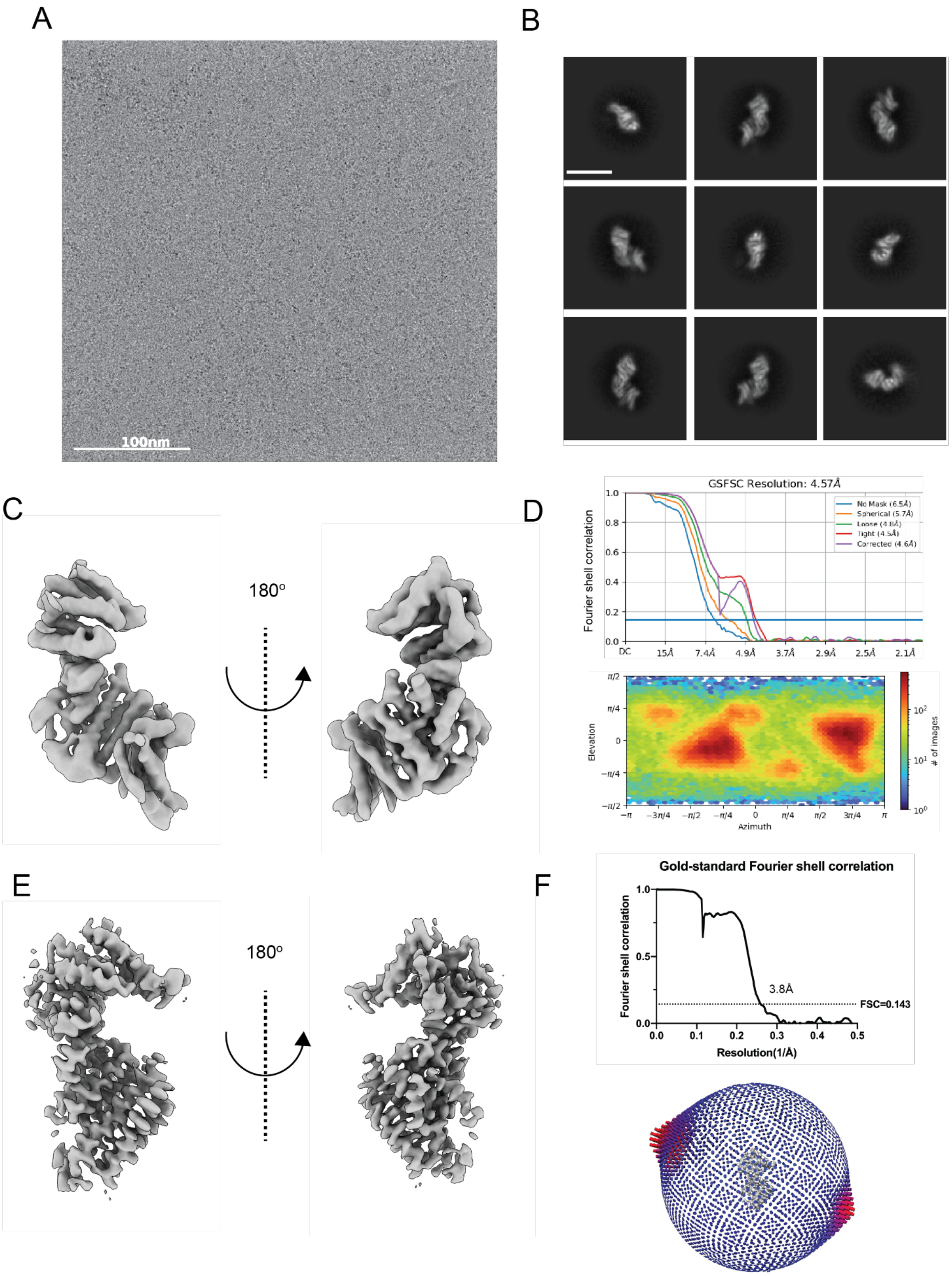

Figure S1. Cryo-EM structures of KIFBP.
(A) Representative cryo-EM micrograph. (B) Representative 2D class averages. Scale bar is 100Å. Full KIFBP reconstruction (C), FSC curves and Euler angle distribution (D). KIFBP core overview (E), FSC curves and Euler angle distribution (F).

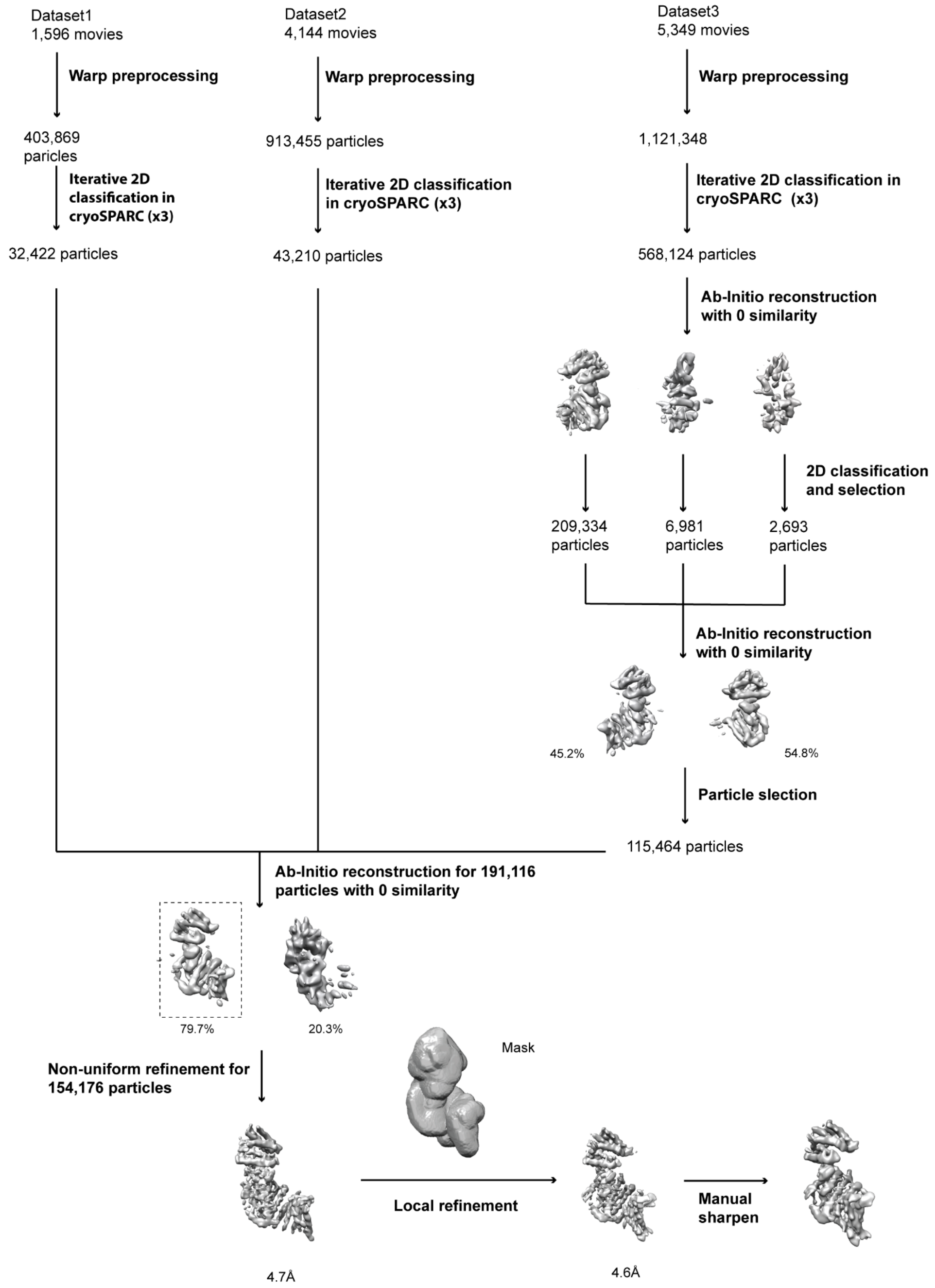

Figure S2. Cryo-EM processing tree for 4.6Ǻ full KIFBP reconstruction.
Overview of data processing strategy for full KIFBP.

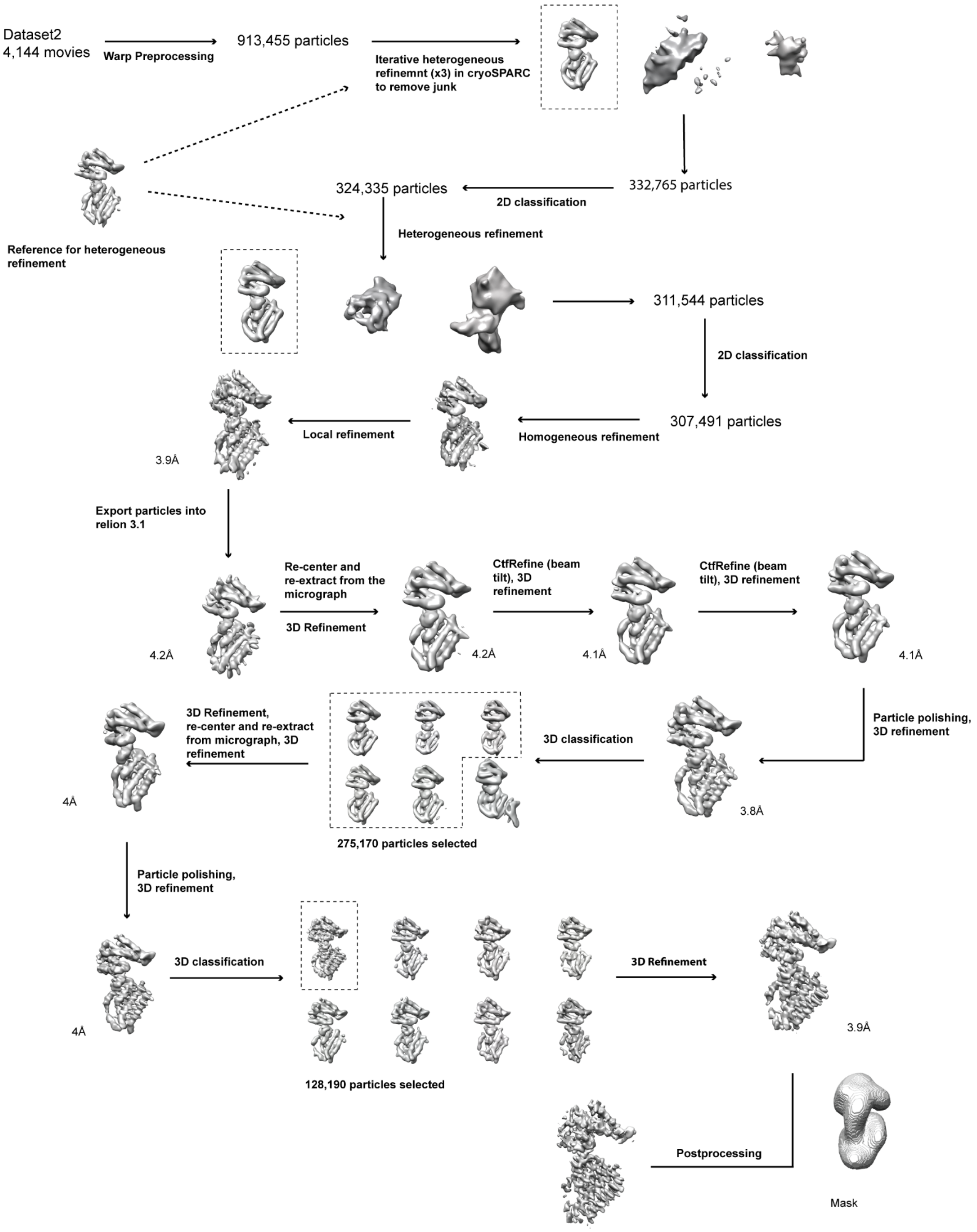

Figure S3. Cryo-EM processing tree for 3.8Å core KIFBP reconstruction. Overview of data processing strategy for core KIFBP reconstruction.

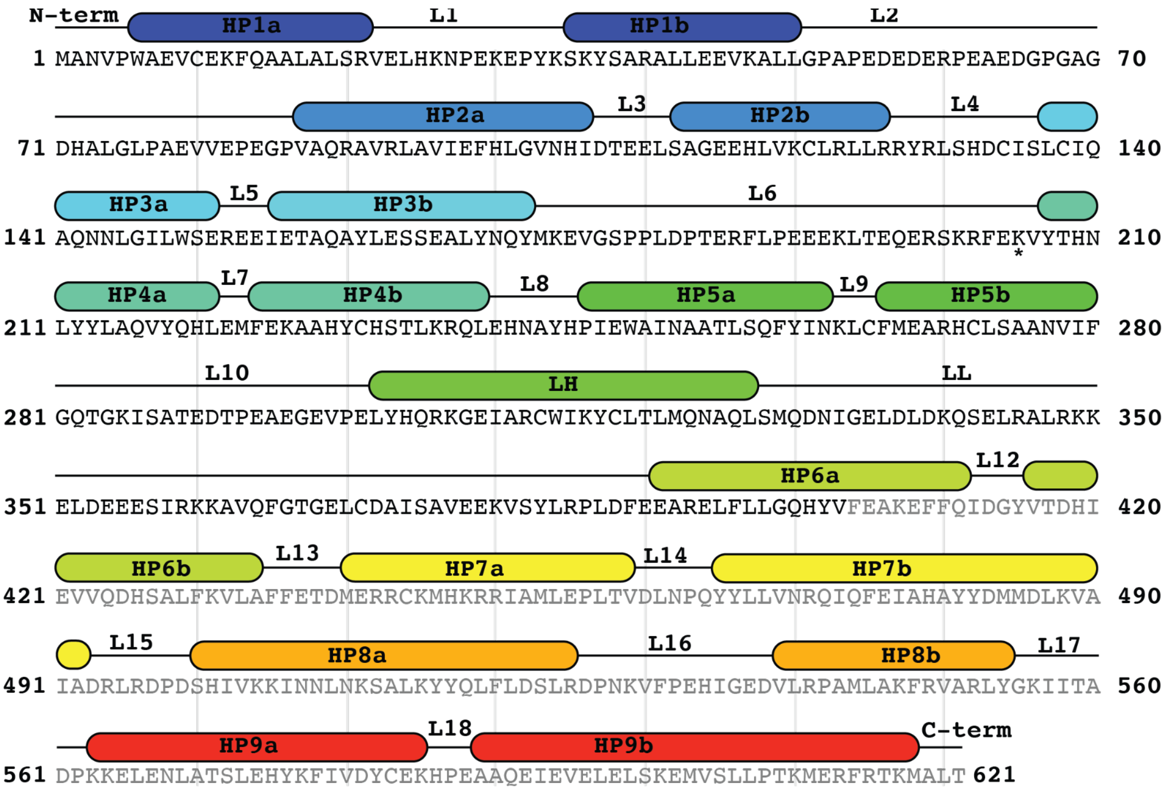

Figure S4. Annotated KIFBP primary sequence using secondary structure information from the atomic model.

Residues 5-403 were built *de novo* into the KIFBP core reconstruction at 3.8Å (black letters), whereas 404-621 were modeled into the 4.6Å reconstruction (gray letters).

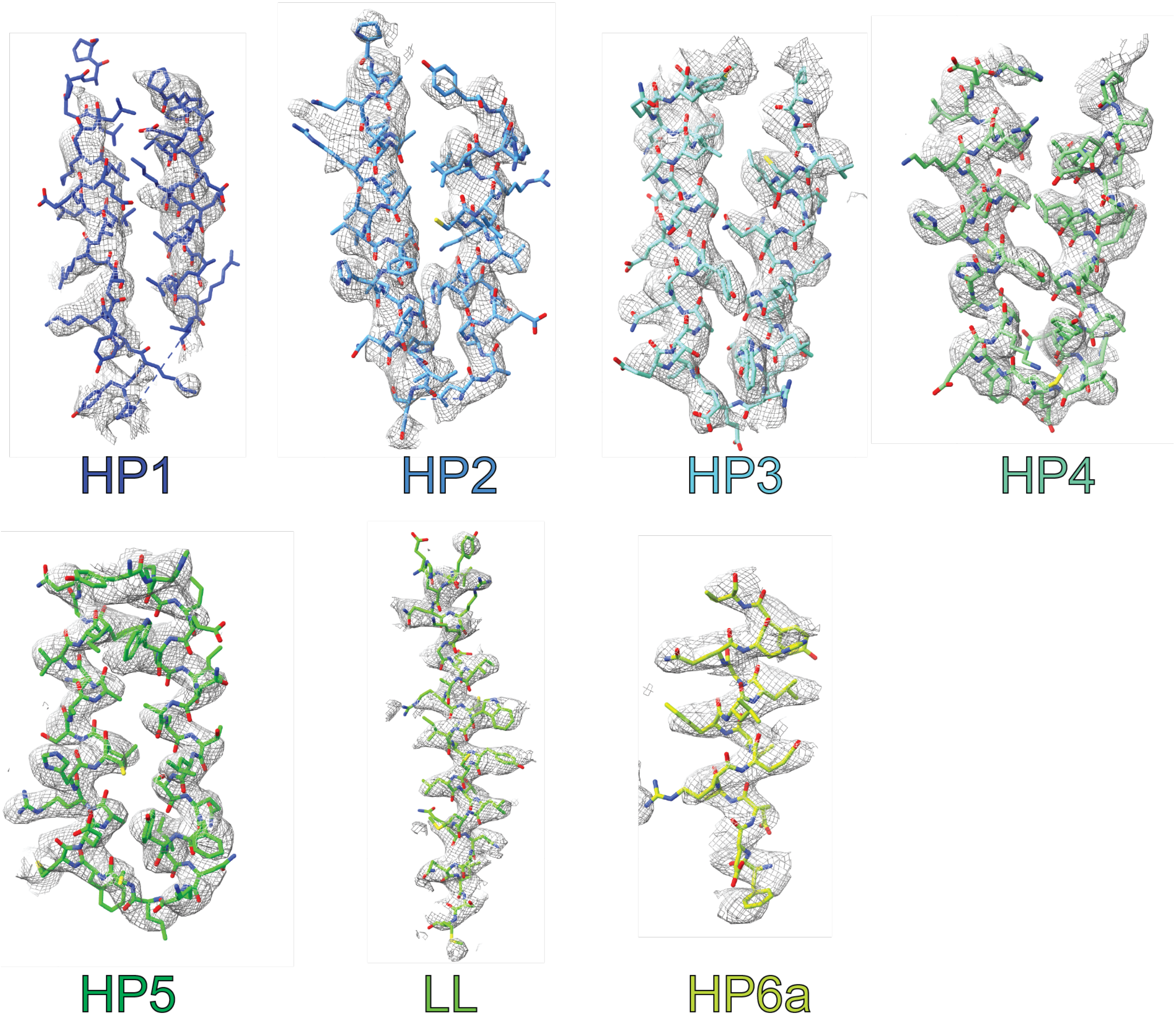

Figure S5. Segmented density for 3.8Å core KIFBP reconstruction and atomic model.
Helical pairs from core KIFBP reconstruction.

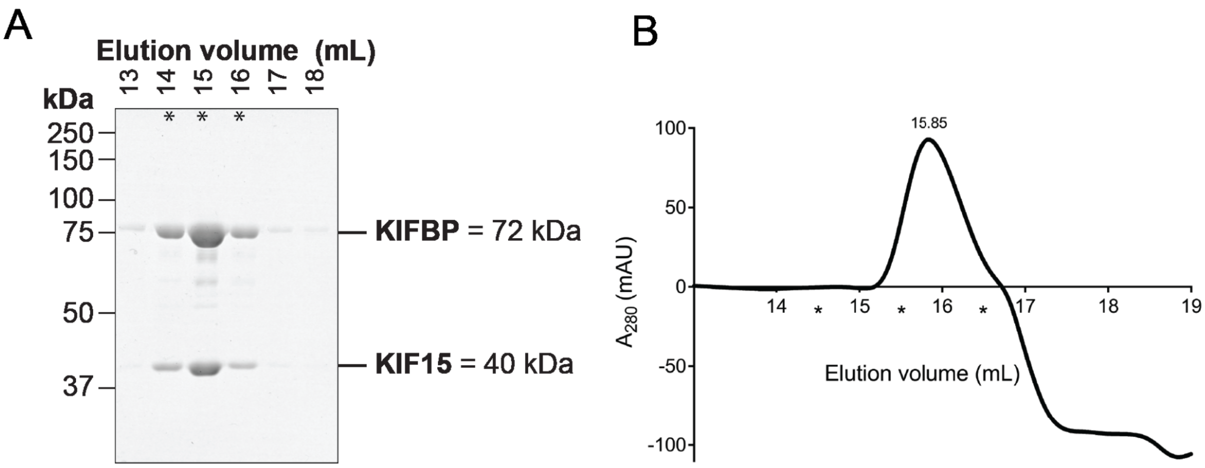

Figure S6. Size exclusion chromatography of KIFBP:KIF15.
KIFBP and KIF15 were purified, combined, and run over the Superose 6 column as described in methods. (A) Representative Coomassie-stained SDS-PAGE of peak fractions from elution profile shown in (B). Fractions 14, 15, and 16 were combined and used in subsequent cryo-EM experiments (indicated by asterisks). The molecular weight of each protein is indicated in kilodaltons.

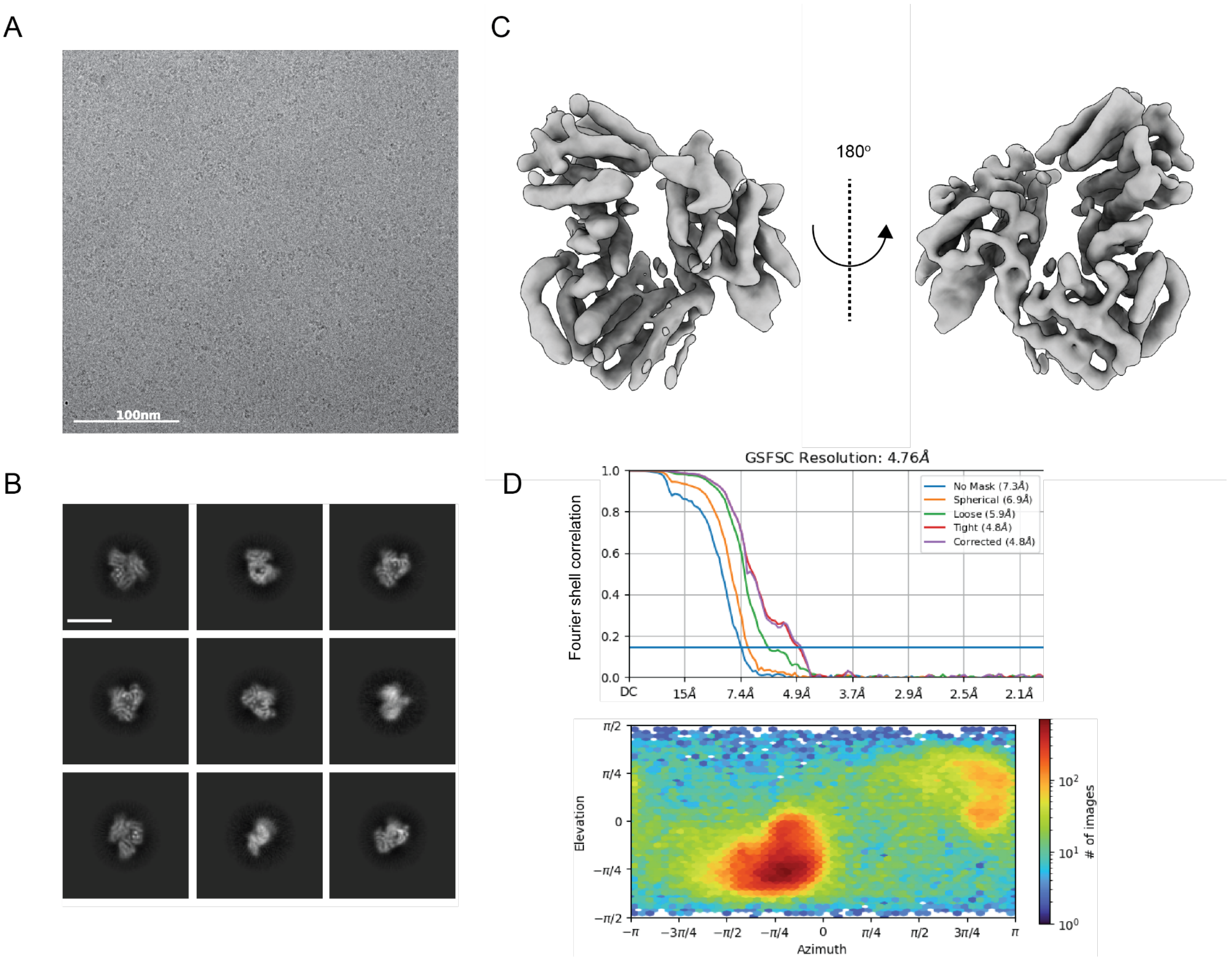

Figure S7. Cryo-EM structure of KIFBP:KIF15.
(A) Representative micrograph for KIFBP:KIF15. (B) Representative 2D class averages. The scale bar is 100Å. (C) Reconstruction overview. (D) FSC curves and Euler angle distribution.

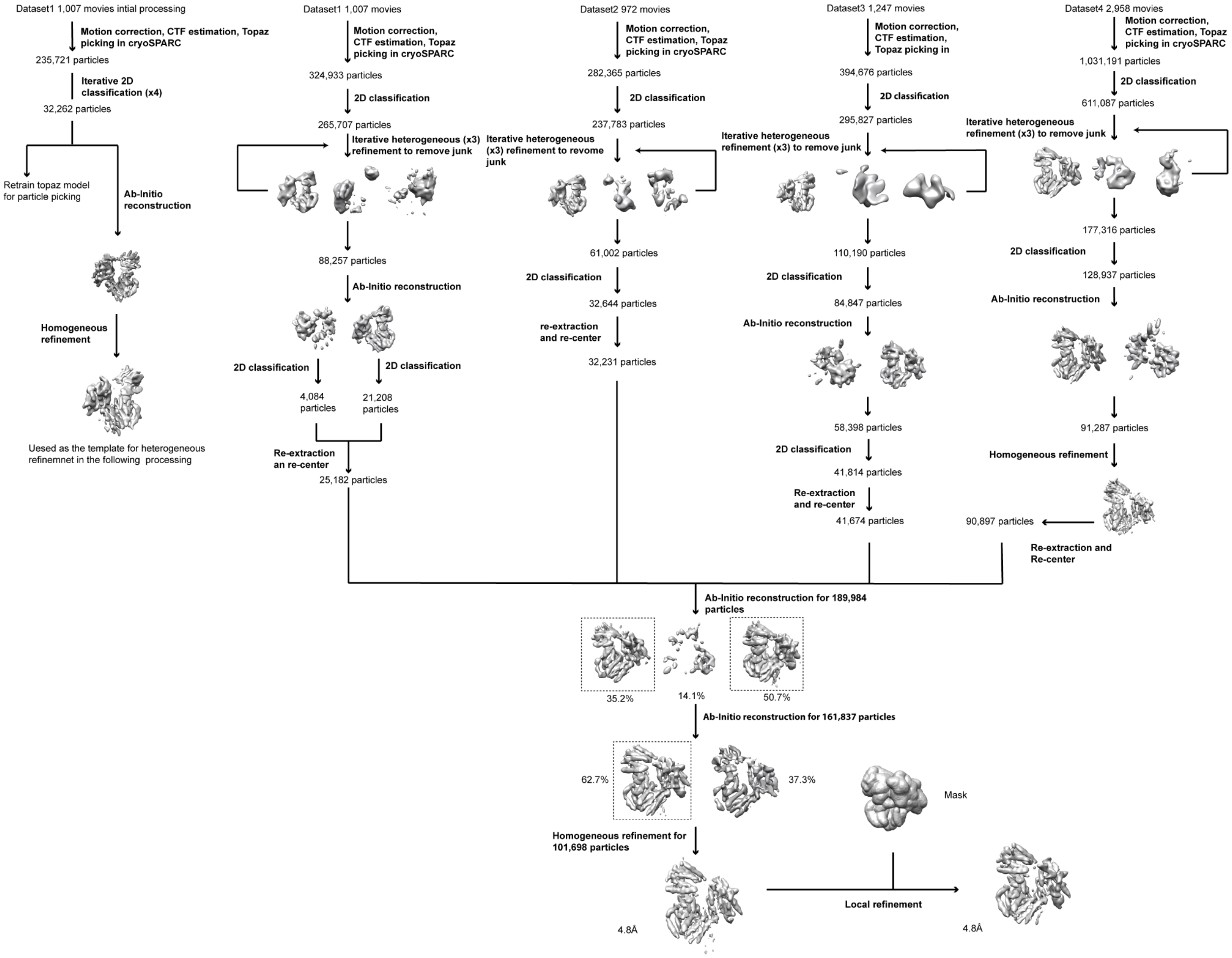

Figure S8. Cryo-EM processing tree for KIFBP:KIF15.
Overview of processing steps for KIFBP:KIF15.

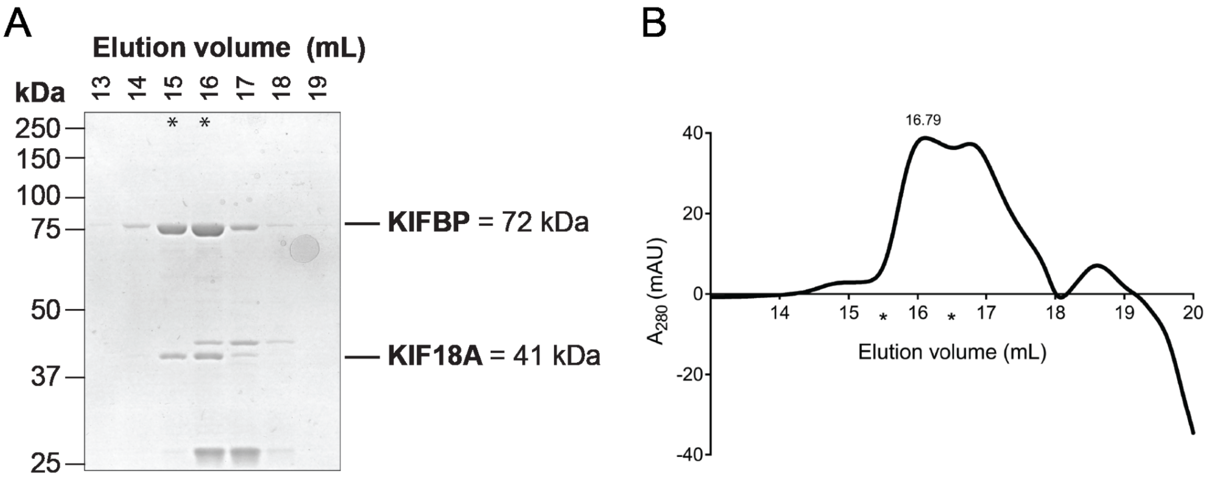

Figure S9. Size exclusion chromatography of KIFBP:KIF18A.
KIFBP and KIF18A were purified, combined, and run over the Superose 6 column as described in methods. (A) Representative Coomassie-stained SDS-PAGE of peak fractions from elution profile shown in (B). Fractions 15 and 16 were combined and used in subsequent cryo-EM experiments (indicated by asterisks). The molecular weight of each protein is indicated in kilodaltons.

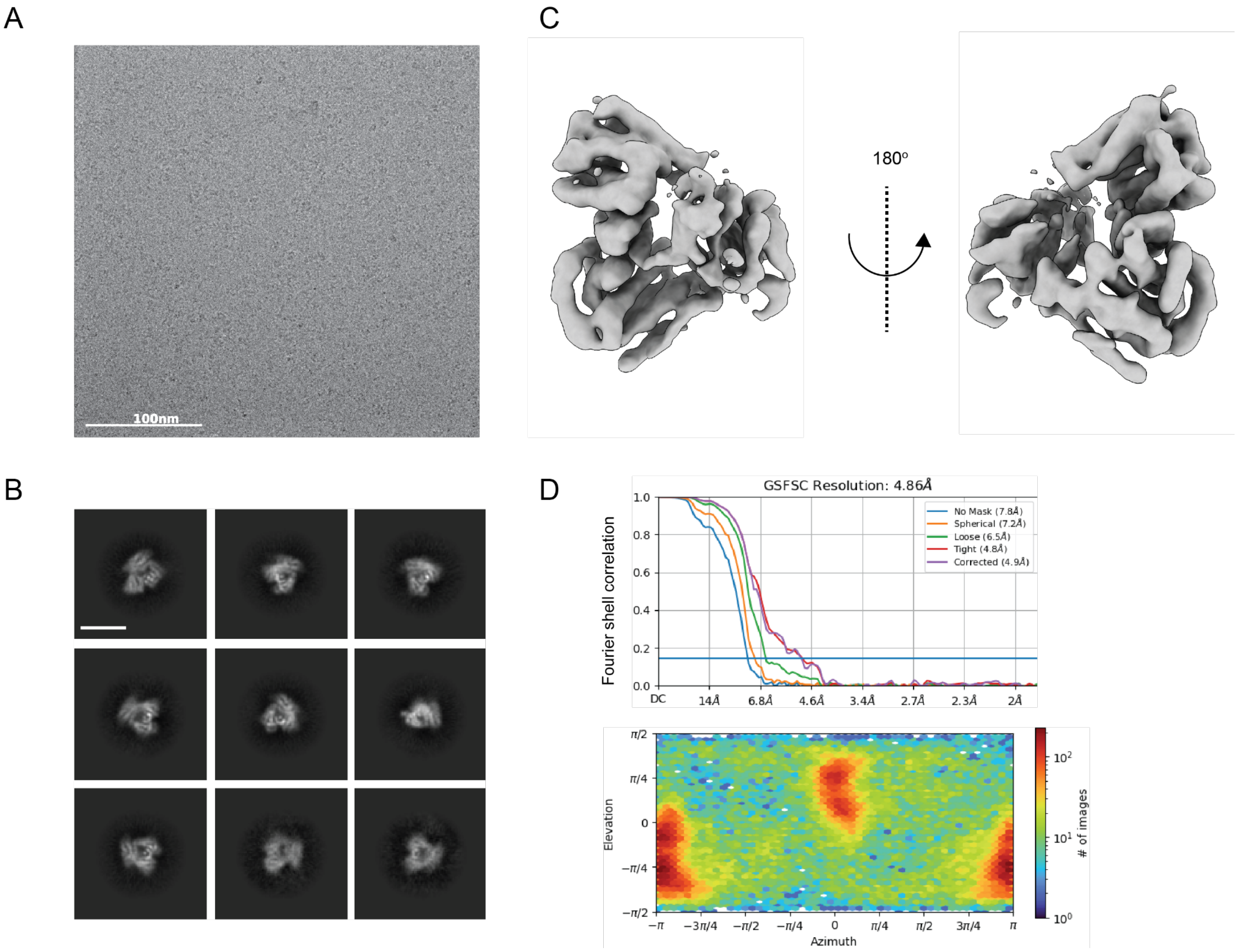

Figure S10. Cryo-EM structure of KIFBP:KIF18A.
(A) Representative micrograph for KIFBP:KIF18A. (B) Representative 2D class averages. Scale bar is 100Å. (C) Reconstruction overview. (D) FSC curves and Euler angle distribution.

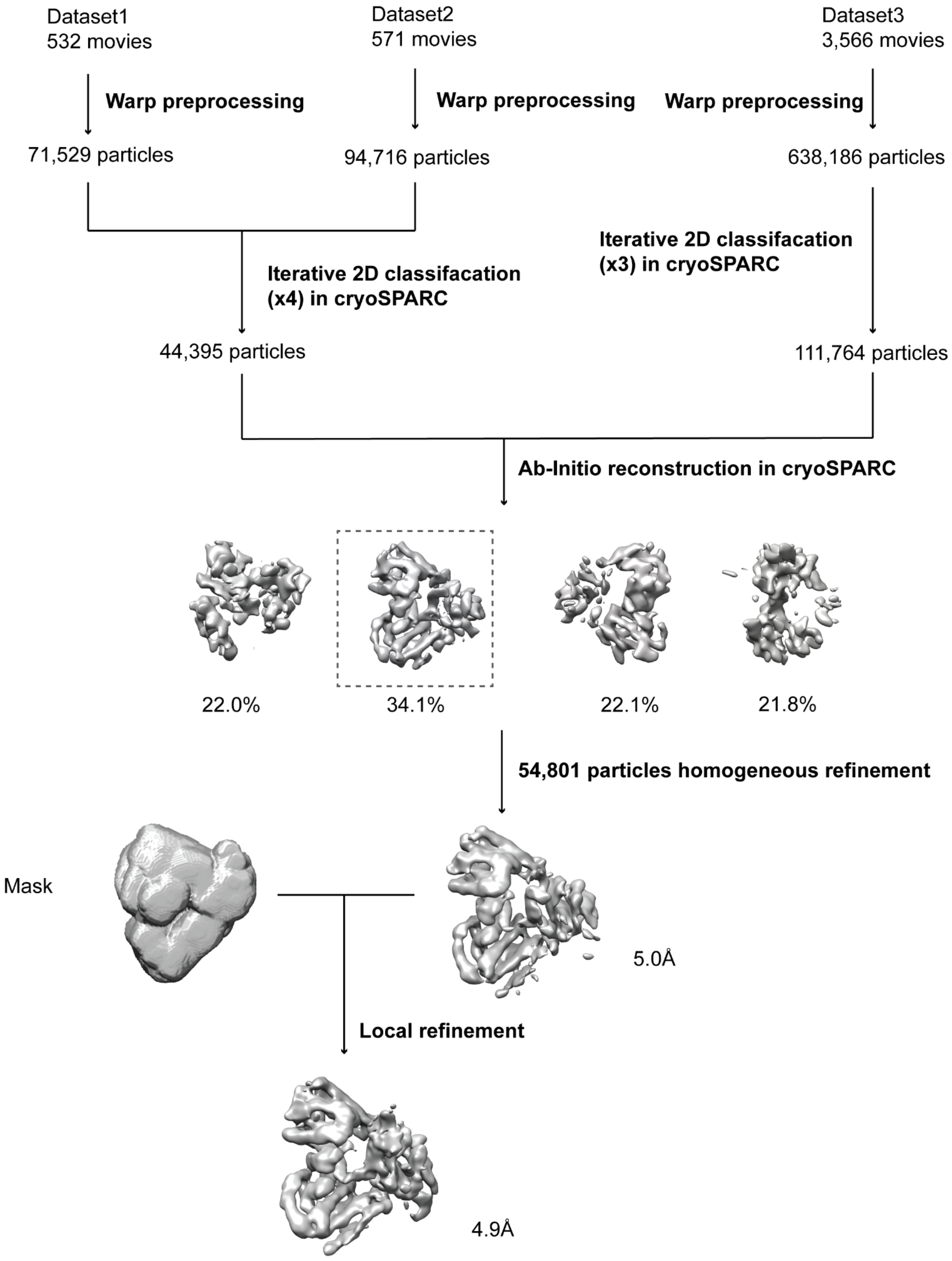

Figure S11. Cryo-EM processing tree for KIFBP:KIF18A.
Overview of analysis strategy for KIFBP:KIF18A.

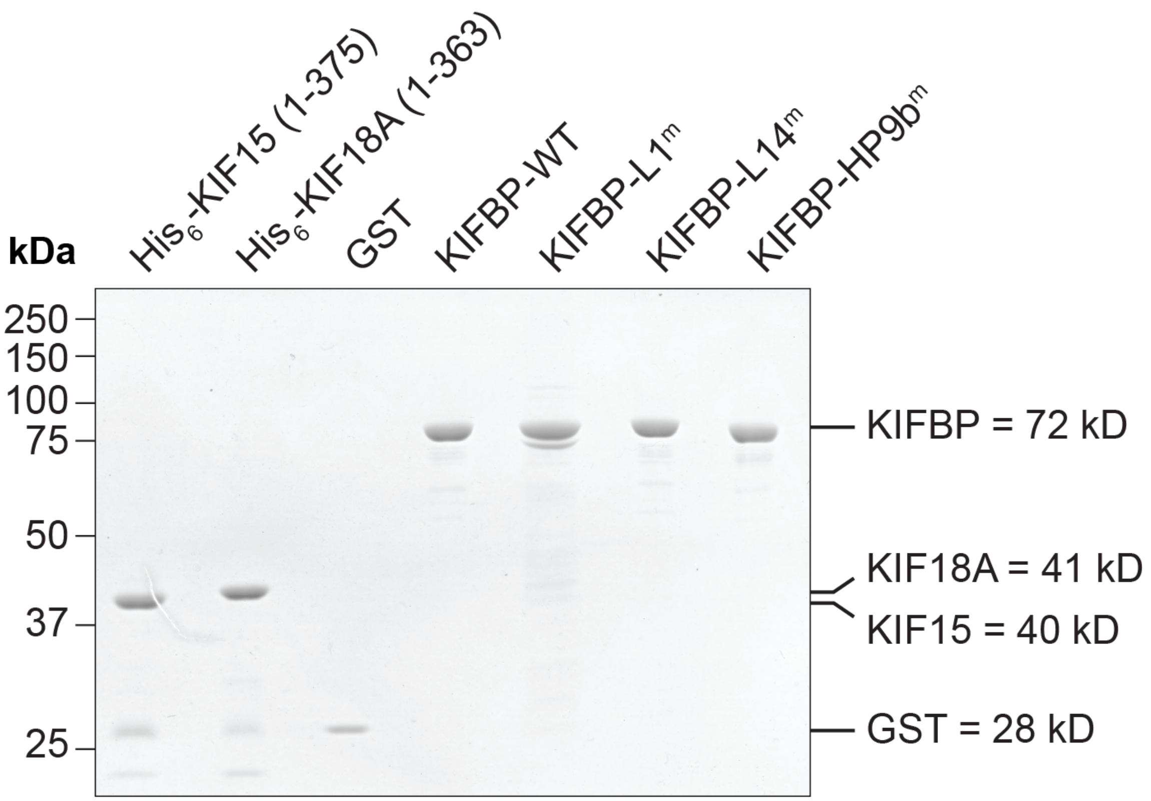

Figure S12. Input of individual proteins used in the pull-down binding assay.
Representative Coomassie gel of 1 µg of each protein used in the pull-down assays in Figure 6C & D.

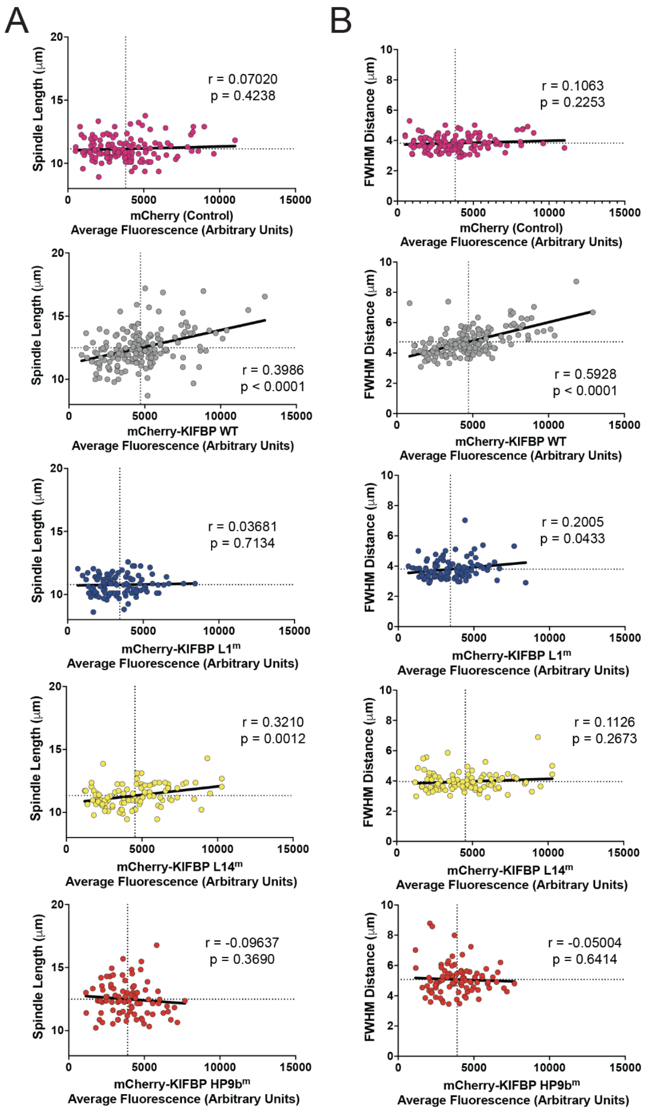

Figure S13. Mitotic effects of mCherry-KIFBP-WT scale with expression level.
(A) Plots of Spindle Length versus Average mCherry Fluorescence for HeLa Kyoto cells overexpressing mCherry or indicated mCherry-KIFBP construct. Each dot represents a single cell. Data presented from a minimum of three independent experiments. Dotted lines represent the mean value for Spindle Length or Average mCherry Fluorescence. Solid line is a linear regression showing the trend of the data. The Pearson's correlation coefficient (r) and two-tailed p-value with 95% confidence interval are shown for each plot. (B) Plots of Full-Width at Half Maximum (FWHM) Distance versus Average mCherry Fluorescence for HeLa Kyoto cells overexpressing mCherry or indicated mCherry-KIFBP construct. Each dot represents a single cell. Data presented from a minimum of three independent experiments. Dotted lines represent the mean value for FWHM distance or Average mCherry Fluorescence. Solid line is a linear regression showing the trend of the data. The Pearson's correlation coefficient (r) and two-tailed p-value with 95% confidence interval are shown for each plot.

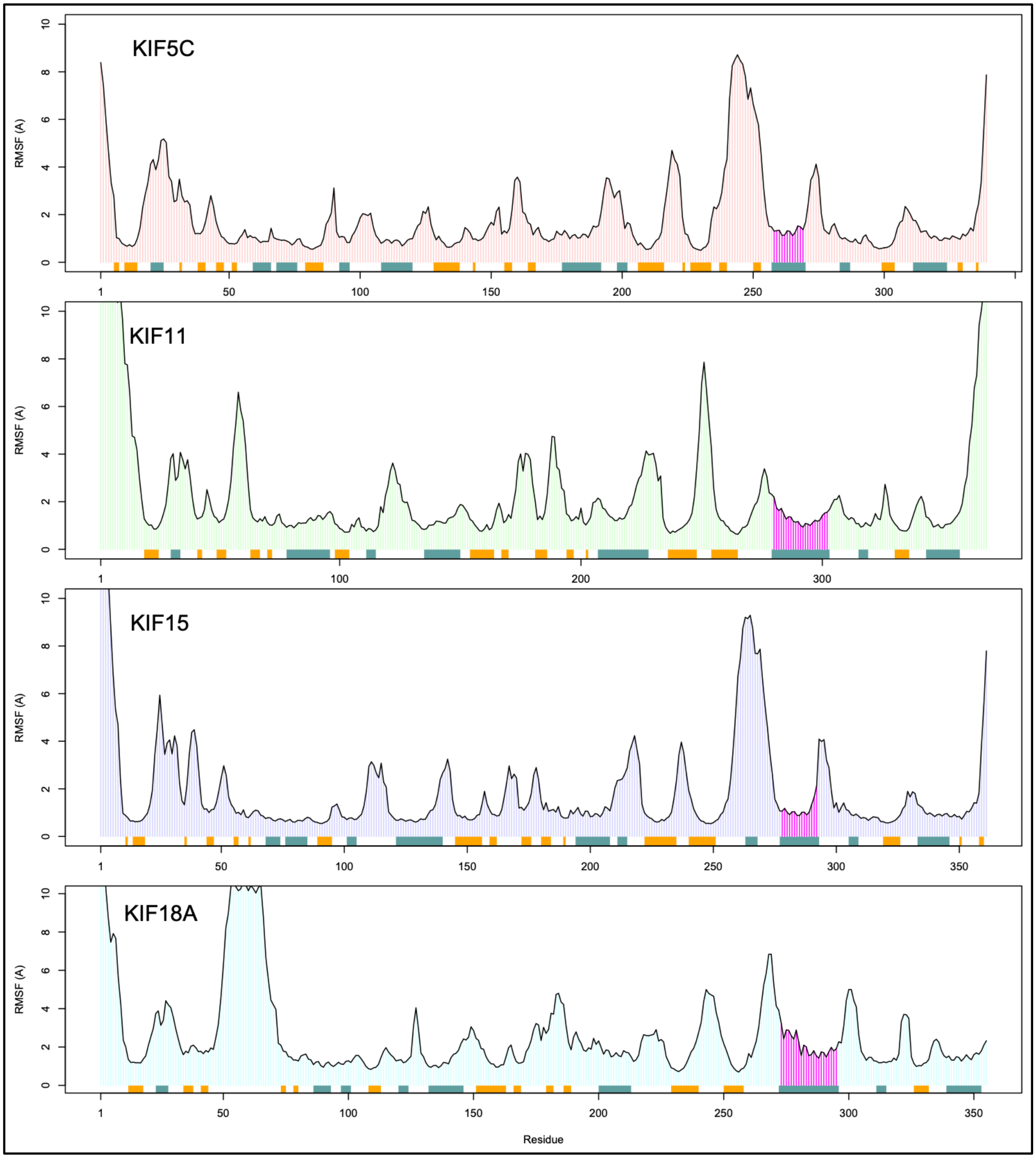

Figure S14. Comparison of structural fluctuations for kinesin motors in solution as measured by MD.

Shown are root mean squared fluctuations (RMSF) for kinesin motor domains in solution. The ribbon at the bottom of each plot shows the secondary structure - green indicates α-helices and orange represents β-strands. The position of α4 is marked by magenta for each protein.

Table S1. Cryo-EM data collection, analysis, and validation statistics for KIFBP (full).

	Structure: KIFBP (full) (EMD-24745) (PDB 7RYQ)		
**Data collection**			
Grids	UltrAuFoil	UltrAuFoil	UltrAuFoil
Vitrification method	FEI Vitrobot	FEI Vitrobot	FEI Vitrobot
Microscope	Glacios	Glacios	Glacios
Session name	20jul13c	20jul07a	20jul11b
Magnification	45000X	45000X	45000x
Voltage (kV)	200	200	200
Stage tilt (°)	0	0	0
Detector	K2 Summit	K2 Summit	K2 Summit
Recording mode	Counting	Counting	Counting
Total electron exposure ($e^{-}/Å^2$)	58.5	68.4	65.0
Number of frames	45	40	35
Defocus range (µm)	0.8 – 2.0	0.8 – 2.0	0.8-2.0
Pixel size (Å)	0.98	0.98	0.98
**Data processing**			
Number of micrographs	5,349	1,596	4,144
Initial particle images (no.)	1,121,348	403,869	913,455
Final particle images (no.)	115,464	32,422	43,210
Final particles in refinement	154,176		
Symmetry	C1		
Map resolution (Å)	4.6		
**Refinement**			
Initial model used (PDB code)	N/A		
Cryo-efficiency (cryoEF)	0.7		
Model resolution (Å)	4.6		
FSC threshold	0.143		
Map sharpening *B* factor ($Å^2$)	-50		
Model composition			
Non-hydrogen atoms	4332		
Protein residues	527		
Ligands	0		
B factors ($Å^2$)	N/A		
Protein			
Ligand			
R.m.s. deviations	N/A		
Bond lengths (Å)			
Bond angles (°)			
Validation	N/A		
MolProbity score			
Clashscore			
Poor rotamers (%)			
Ramachandran plot	N/A		
Favored (%)			
Allowed (%)			
Disallowed (%)			

Table S2. Cryo-EM data collection, analysis, and validation statistics for KIFBP (core).

	**Structure: KIFBP (core)**<br>(EMD-24677)<br>(PDB 7RSQ)
**Data collection**	
Grids	UltrAuFoil
Vitrification method	FEI Vitrobot
Microscope	Glacios
Session name	20jul11b
Magnification	45000X
Voltage (kV)	200
Stage tilt (°)	0
Detector	K2 Summit
Recording mode	Counting
Total electron exposure ($e^-/Å^2$)	65
Number of frames	35
Defocus range (μm)	0.8 – 2.0
Pixel size (Å)	0.98
**Data processing**	
Number of micrographs	4,144
Initial particle images (no.)	913,455
Final particles in refinement	128,190
Symmetry	C1
Map resolution (Å)	3.8
**Refinement**	
Cryo-efficiency (cryoEF)	0.5
FSC model to map (0.143, 0.5)	3.5, 4.5
Initial model used (PDB code)	N/A
Model resolution (Å)	3.8
FSC threshold	0.143
Map sharpening *B* factor ($Å^2$)	-50
Model composition	
Non-hydrogen atoms	2015
Protein residues	246
Ligands	0
B factors ($Å^2$)	
Protein	57.76
Ligand	N/A
R.m.s. deviations	
Bond lengths (Å)	0.008
Bond angles (°)	0.942
Validation	
MolProbity score	1.65
Clashscore	3.25
Poor rotamers (%)	2.88
Ramachandran plot	
Favored (%)	96.90
Allowed (%)	3.10
Disallowed (%)	0

Table S3. Cryo-EM data collection, analysis, and validation statistics for KIFBP:KIF15.

	**Structure: KIFBP:KIF15** (EMD-24744) (PDB 7RYP)			
**Data collection**				
Grids	UltrAuFoil	UltrAuFoil	UltrAuFoil	UltrAuFoil
Vitrification method	FEI Vitrobot	FEI Vitrobot	FEI Vitrobot	FEI Vitrobot
Microscope	Glacios	Glacios	Glacios	Glacios
Session name	20aug23f	20aug30b	20sep17h	20sep25f
Magnification	45000X	45000X	45000X	45000X
Voltage (kV)	200	200	200	200
Stage tilt (°)	0	0	0	0
Detector	K2 Summit	K2 Summit	K2 Summit	K2 Summit
Recording mode	Counting	Counting	Counting	Counting
Total electron exposure (e^-/Å^2)	83.6	60.1	61.25	60.76
Number of frames	40	30	35	35
Defocus range (μm)	0.8 – 2.0	0.8 – 2.0	0.8-2.0	0.8-2.0
Pixel size (Å)	0.98	0.98	0.98	0.98
**Data processing**				
Number of micrographs	1,007	972	1,247	2,958
Initial particle images (no.)	324,933	282,365	394,676	1,031,191
Final particle images (no.)	25,182	32,231	41,674	90,897
Final particles in refinement	101,698			
Symmetry	C1			
Map resolution (Å)	4.8			
**Refinement**				
Initial model used (PDB code)	4BN2			
Cryo-efficiency (cryoEF)	0.58			
Model resolution (Å)	4.8			
FSC threshold	0.143			
FSC model to map (0.143, 0.5)	4.8, 7.1			
Map sharpening *B* factor (Å^2)	-200			
Model composition				
Non-hydrogen atoms	5699			
Protein residues	725			
Ligands	0			
B factors (Å^2)				
Protein	199			
Ligand	0			
R.m.s. deviations				
Bond lengths (Å)	0.015			
Bond angles (°)	1.382			
Validation				
MolProbity score	1.68			
Clashscore	4.15			
Poor rotamers (%)	0			
Ramachandran plot				
Favored (%)	92.08			
Allowed (%)	6.22			
Disallowed (%)	1.70			

Table S4. Cryo-EM data collection, analysis, and validation statistics for KIFBP:KIF18A.

	**Structure: KIFBP:KIF18A** (EMD-24672) (PDB 7RSI)		
**Data collection**			
Grids	UltrAuFoil	UltrAuFoil	UltrAuFoil
Vitrification method	FEI Vitrobot	FEI Vitrobot	FEI Vitrobot
Microscope	Talos Arctica	Talos Arctica	Talos Arctica
Session name	20oct06g	20nov05c	21jan22b
Magnification	45000X	45000X	45000x
Voltage (kV)	200	200	200
Stage tilt (°)	0	0	0
Detector	K2 Summit	K2 Summit	K2 Summit
Recording mode	Counting	Counting	Counting
Total electron exposure ($e^{-}/Å^2$)	57.0	51.8	61.2
Number of frames	35	35	40
Defocus range (μm)	0.8 – 2.0	0.8 – 2.0	0.8-2.0
Pixel size (Å)	0.91	0.91	0.91
**Data processing**			
Number of micrographs	532	571	3,566
Initial particle images (no.)	71,529	94,716	638,186
Final particle images (no.)	44,395		111,764
Final particles in refinement	54,801		
Symmetry	C1		
Map resolution (Å)	4.9		
**Refinement**			
Initial model used (PDB code)	3LRE		
Cryo-efficiency (cryoEF)	0.67		
Model resolution (Å)	4.9		
FSC threshold	0.143		
Map sharpening *B* factor ($Å^2$)	-163.5		
Model composition	N/A		
Non-hydrogen atoms			
Protein residues			
Ligands			
B factors ($Å^2$)	N/A		
Protein			
Ligand			
R.m.s. deviations	N/A		
Bond lengths (Å)			
Bond angles (°)			
Validation	N/A		
MolProbity score			
Clashscore			
Poor rotamers (%)			
Ramachandran plot	N/A		
Favored (%)			
Allowed (%)			
Disallowed (%)			

Table S5. High-confidence crosslinks between KIFBP and KIF15.
A summary of all high-confidence crosslinks identified between KIFBP and the KIF15 motor domain using mass spectrometry and the lysine-targeting crosslinker BS3. The position of KIFBP and KIF15 residues of each crosslink are shown.

KIFBP Residue	KIF15 Residue
26	273
	283
	319
	361
30	273
	283
	319
	361
	364
	366
36	273
	283
205	273
	283
307	283
	319
350	364
556	361
	366
564	283
	364
610	319
	361
	364
	366
617	361
	364

Movie S1. Summary video describing the mechanism of kinesin inhibition by KIFBP.
